# FGF2 promotes the expansion of parietal mesothelial progenitor pools and inhibits BMP4-mediated smooth muscle cell differentiation

**DOI:** 10.3389/fcell.2024.1387237

**Published:** 2024-09-23

**Authors:** Youngmin Hwang, Yuko Shimamura, Junichi Tanaka, Akihiro Miura, Anri Sawada, Hemanta Sarmah, Dai Shimizu, Yuri Kondo, Hyeonjeong Lee, Francesca Martini, Zurab Ninish, Kelley S. Yan, Kazuhiko Yamada, Munemasa Mori

**Affiliations:** ^1^ Columbia Center for Human Development (CCHD), Columbia University Irving Medical Center, New York, NY, United States; ^2^ Department of Medicine, Division of Digestive and Liver Diseases, Columbia University Irving Medical Center, New York, NY, United States; ^3^ Department of Genetics and Development, Columbia University Irving Medical Center, New York, NY, United States; ^4^ Herbert Irving Comprehensive Cancer Center, Columbia University Irving Medical Center, New York, NY, United States; ^5^ Department of Surgery, School of Medicine, Johns Hopkins Medicine, Baltimore, MD, United States

**Keywords:** parietal mesothelial cell self-renewal, differentiation, maturation, FGF2, BMP4, PDGF, wnt

## Abstract

Mesothelial cells, in the outermost layer of internal organs, are essential for both organ development and homeostasis. Although the parietal mesothelial cell is the primary origin of mesothelioma that may highjack developmental signaling, the signaling pathways that orchestrate developing parietal mesothelial progenitor cell (MPC) behaviors, such as MPC pool expansion, maturation, and differentiation, are poorly understood. To address it, we established a robust protocol for culturing WT1^+^ MPCs isolated from developing pig and mouse parietal thorax. Quantitative qPCR and immunostaining analyses revealed that BMP4 facilitated MPC differentiation into smooth muscle cells (SMCs). In contrast, FGF2 significantly promoted MPC progenitor pool expansion but blocked the SMC differentiation. BMP4 and FGF2 counterbalanced these effects, but FGF2 had the dominant impact in the long-term culture. A Wnt activator, CHIR99021, was pivotal in MPC maturation to CALB2^+^ mesothelial cells, while BMP4 or FGF2 was limited. Our results demonstrated central pathways critical for mesothelial cell behaviors.

## 1 Introduction

The mesothelium, a distinctive cell type forming the pleural monolayer, envelopes the outermost layers of the viscera and facilitates the growth of developing organs. Despite the known fact that aberrant proliferation of adult mesothelial cells, often aggravated by asbestos exposure, can lead to mesothelioma through the manipulation of developmental pathways, the specific signaling processes that dictate progenitor pool expansion, embryonic mesothelial progenitor cell (MPC) maturation, and their differentiation into smooth muscle cells (SMC) remain poorly understood.

Anatomically, adult mature mesothelial cells of the parietal and visceral pleura encase the inner layer of the thorax and the outer layer of the lungs, respectively. Mouse lineage-tracing analyses showed that visceral mesothelial cells in developing lung pleura migrate inward and differentiate into vascular smooth muscle cells (Que et al., 2008; Cano et al., 2013), and parabronchial smooth muscle cells (De Langhe et al., 2008), highlighting the multipotency of developmental MPCs. During development, the MPC arises from the exact origin, lateral plate mesoderm (Obacz et al., 2021), while mesothelioma tends to originate from parietal mesothelial cells (Boutin et al., 1998). Since carcinogenesis often hijacks developmental programs (Manzo, 2019), studying parietal mesothelial development could significantly advance mesothelioma diagnosis and treatment.

Mesothelioma, a rare and aggressive cancer often caused by carcinogens like asbestos or tar, has a notably high mortality rate (Rehrauer et al., 2018). The prevalence is high in the countries such as the United Kingdom, Australia, and New Zealand (Huang et al., 2023). Various tumor markers were identified, including calretinin (CALB2), mesothelin (MSLN), type III collagen (COL3A1), and secretory leukocyte peptidase inhibitor (SLP1) (Gueugnon et al., 2011). Despite the availability of treatments such as surgical decertification and chemotherapy, most cases are diagnosed at advanced stages, limiting effective intervention options (Ricciardi et al., 2018). A better understanding of the behavior of MPCs in the parietal pleura during development could develop the prognostic markers of mesothelioma.

In mouse embryos, wilms tumor protein 1 (WT1), a representative mesothelial cell marker, is expressed on visceral and parietal mesothelial cells from the lung and the thoracic cavity (Que et al., 2008; Cano et al., 2013). WT1 knockout mice showed hypoplastic lung phenotype (Cano et al., 2013; Sontake et al., 2018) and the defects of human mesothelial cells by congenital diaphragmatic hernia (CDH), also known to develop lung hypoplasia (Gilbert et al., 2021).

Previous *in vitro* studies have shown that Fibroblast growth factor 2 (FGF2) and platelet-derived growth factor (PDGF) are required for the proliferation of adult mesothelial cells (Mutsaers et al., 1997). Notably, high expression of FGF2 in mesothelioma correlates with poor prognosis (Kumar-Singh et al., 1999).

Bone morphogenic protein 4 (BMP4) is expressed in the human adult peritoneal mesothelium and plays a pivotal role in mesothelial-to-mesenchymal transition (MMT), attenuating the TGF-beta-mediated MMT phenotype (Namvar et al., 2018). BMP4 is expressed ventral to the distal lung bud mesenchyme and at the distal lung bud tips of the endoderm (Weaver et al., 1999; Weaver et al., 2000), but the association with the behavior of WT1^+^ MPC is unknown.

Additionally, sonic hedgehog (SHH) and retinoic acid (RA) are implicated in MPC migration and epithelial morphology transformation, respectively (Dixit et al., 2013).

However, how these signaling pathways intertwine and distinctively regulate MPC pool expansion, differentiation, and maturation during development has yet to be determined, necessitating robust culture methods for detailed study.

This study successfully allowed us to establish the method to isolate and culture embryonic parietal MPC from developing pig and mouse thorax. By culturing these cells with a range of small molecules and growth factors, we aimed to elucidate the signaling pathways crucial for mesothelial cell development.

## 2 Results

### 2.1 Establishment of cell culture protocol for the expansion of developing pig mesothelial cells

The development of pig lungs undergoes embryonic, pseudo glandular, canalicular, and alveolar stages around embryonic day 19 (E19), E25, E60, and E90, respectively (McGeady et al., 2017; Shimamura et al., 2022). The developmental stage at which pig parietal mesothelial progenitor cells (MPCs) could be efficiently harvested was unknown. We harvested the parietal MPCs from the E80 canalicular stage thorax to have enough cell numbers.

**FIGURE 1:**
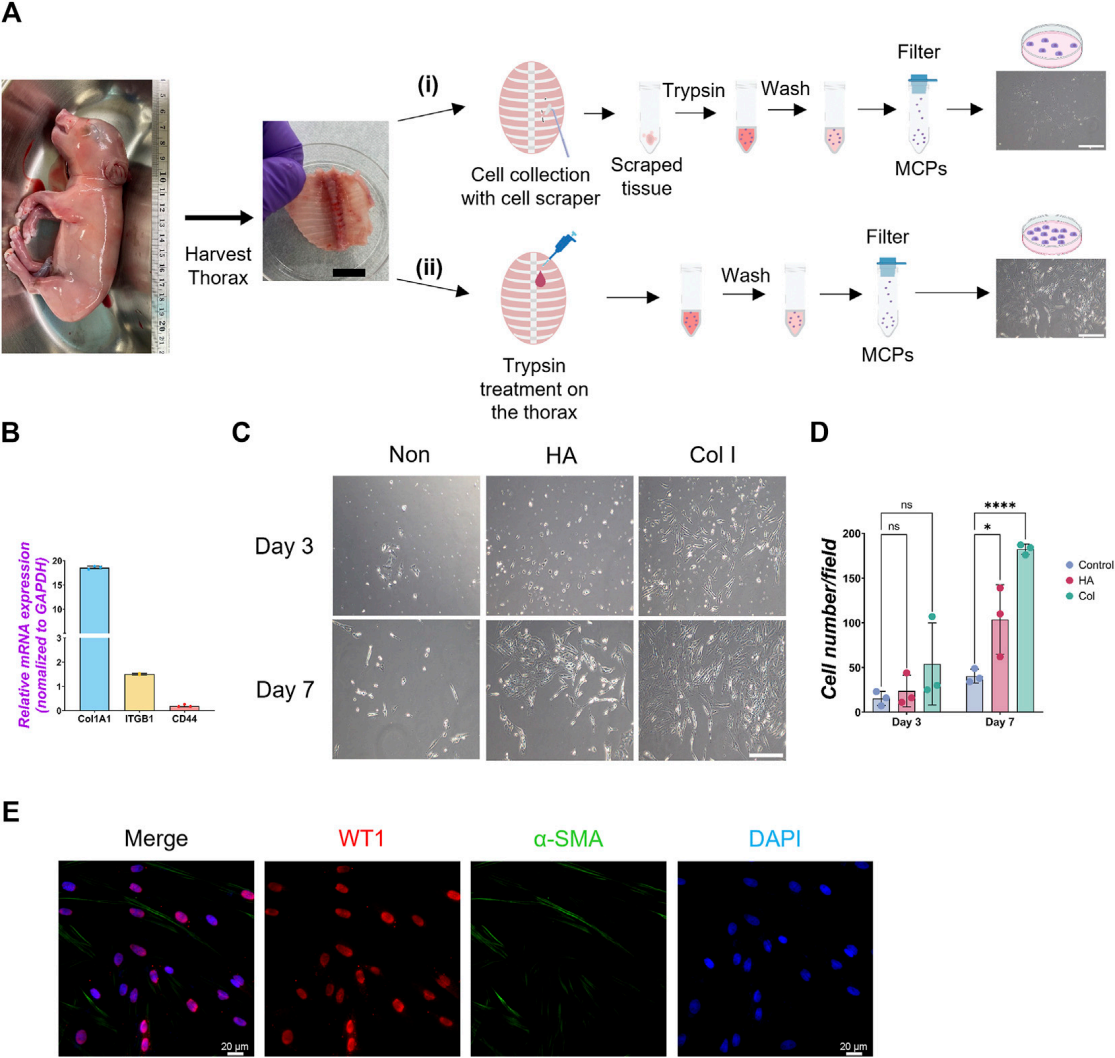
Isolation of mesothelial cell progenitors (MPCs) from pig fetuses. **(A)** Schematic illustration of pig MPC isolation: The embryonic thorax (middle panel in A) was isolated from E80 pig fetuses (left panel in A) and treated with the following procedures. **(i)** Scraping MPCs followed by trypsinization with 0.05% trypsin in the tube: **(ii)** trypsinization with 0.05% trypsin directly on the thorax. In both methods, the mesothelial cell was neutralized with DMEM +10% FBS, followed by PBS washing and filtration with a cell strainer to remove the residual connective tissue. The trypsinization on the porcine thorax **(ii)** method showed a higher yield of MPC expansion than the scraping method **(i)** (right panels in A). **(B)** Graph: quantitative qRT-PCR (RT-qPCR) analysis of type I collagen (*COL1A1*), integrin beta-1 (*ITGB1*), and *CD44* cultured in a basal culture medium. Error bars represent mean ± SD. Each plot showed different biological replicates (n = 3). Each gene expression was normalized with the housekeeping gene (*GAPDH*) expression. **(C)** Representative phase contrast images of MPCs isolated from E80 pig thorax cultured on different cell culture dish coating conditions. Col I: type I collagen coating, HA: hyaluronic acid coating, Non: non-coating. **(D)** Graphs: Quantification of the isolated pig MPC number per each field. Each plot showed different biological replicates (n = 3). **(E)** Representative immunofluorescence (IF) image of MPCs after 3 days of culture. Red: WT1, Green: α-SMA, Blue: DAPI. Scale bars: **(A)** 1 cm, **(C)** 100 μm, **(E)** 20 μm. *p < 0.05, ****p < 0.0001, ns: no significant difference by one-way ANOVA test and *t*-test in **(D)**.

To harvest a WT1^+^ developing MPC efficiently, we compared several methods previously reported (Kienzle et al., 2018; Kawai et al., 2019; Pruett et al., 2020; Mierzejewski et al., 2021), including collecting pleural fluid, pinching porcine thoracic walls with tweezers, scaring it with scrapers, or trypsinizing the porcine thoracic wall. Among those methods, trypsinization with a 0.05% trypsin inside the E80 thoracic walls showed the highest yield of MPC collection (Figure 1A). Interestingly, 0.25% trypsin treatment to the thorax did not expand the MPC (Supplementary Figure S1A). Previous papers showed the requirement of EGF for culturing MPCs (Pruett et al., 2020; Mierzejewski et al., 2021). Contrary to expectations, MPC culture with EGF did not offer an apparent effect on MPC colony expansion (Supplementary Figure S1C). To expand MPC efficiently, we coated the cell culture dish with extracellular matrix (ECM) molecules (type I collagen (Col I) and hyaluronic acid (HA)), given their expression in adult mature mesothelial cells (Breborowicz et al., 1996; Saed et al., 1999). We found that the isolated MPC showed the sustained expression of Col I expression and its receptor, integrin beta 1 (ITGB1), but a relatively low expression of HA receptor (CD44) (Figure 1B). Indeed, Col I coating significantly enhanced MPC expansion compared to HA coating (HA) and an uncoated control (Figures 1C, D). Since the gelatin and Col I share the integrin-binding motif RGD sequence (Davidenko et al., 2016), we cultured the MPC on the gelatin-coated dish and confirmed its efficacy in expanding MPCs (Breborowicz et al., 1996), showing WT1^+^ cells (76.7%), α-SMA^+^ cells (7.5%), WT1^−^α-SMA^-^ cells (3.7%), and WT1^−^α-SMA^-^ cells (19.5%) (Figure 1E). These results indicate that most cells are WT1^+^ cells. Based on this, we performed all downstream analyses on the gelatin-coated dish. Additionally, we confirmed that mouse MPC can be collected and expanded well after the trypsinization directly on the E17.5 mouse canalicular ∼ sacculation stage thorax, noting that 0.25% trypsin was more effective for mouse MPCs than 0.05% (Supplementary Figure S1D). To compare the efficacy of MPC isolation with another method, we isolated MPCs from WT1-lineage tracing mice prepared by crossing WT1^CreERT2/+^ x Rosa26^tdTomato/tdTomato^ mice (Supplementary Figure S2). Briefly, we injected Tamoxifen at E15.5 and E16.5 and examined the proportion of tdTomato cells in lung mesenchyme and thorax fraction at E17.5 by FACS analysis. After the exclusion of hematopoietic cells (CD45), epithelial cells (EpCAM), and endothelial cells (PECAM) as previously reported (Miura et al., 2023), tdTomato^+^ cells were sorted from the lung mesenchyme (Supplementary Figure S2B). Compared with lung mesenchyme, the proportion of tdTomato^+^ cells sorted from the thorax surface was relatively higher (2.70% ± 2.14) but insignificant, while the cell number was very low due to the tissue size. The sorted tdTomato^+^ MPCs expressed WT1 after 3 days of culture (Supplementary Figure S2C). However, it was challenging to expand them efficiently due to the low number of sorted cells (Supplementary Figure S2C). Given that we obviously observed more number of the cells in our method (Supplementary Figure S1E), these results underscore the robustness and effectiveness of our trypsinization-based protocol over the sorting-based method for isolating parietal MPCs in development.

### 2.2 FGF2 promotes expansion of pig mesothelial progenitor cells (MPCs)

**FIGURE 2:**
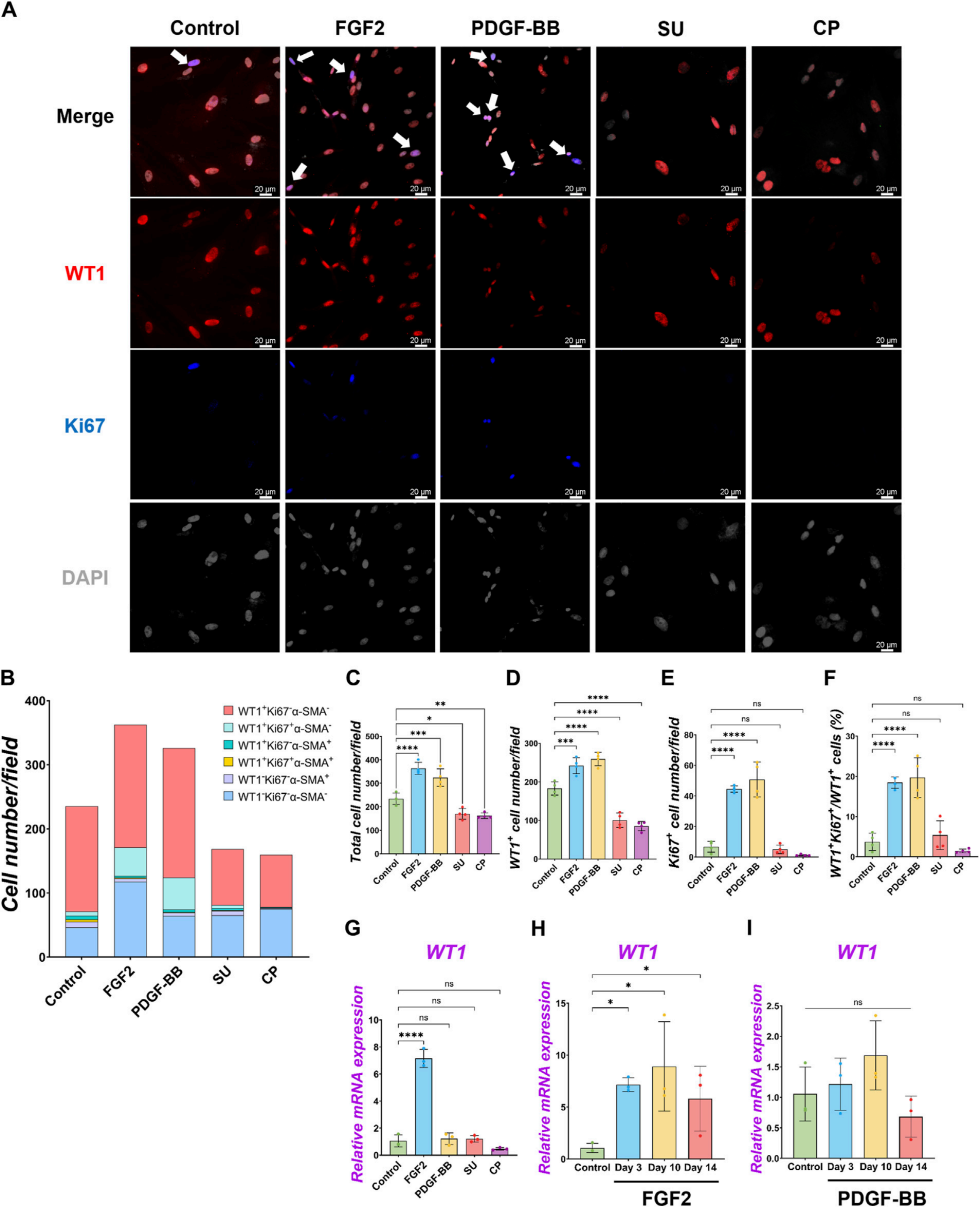
Pig parietal MPC self-renewal by FGF2, and PDGF-BB stimulation. **(A)** Representative IF images of MPCs after 3 days of treatment with FGF2, PDGF, SU5404 (FGF signaling inhibitor, SU), a CP673451 (PDGF signaling inhibitor, CP), or Control (no treatment). FGF2 and PDGF-BB showed more cell numbers per field. WT1 (red), Ki67 (blue), DAPI (grey). Arrows (white): WT1^+^Ki67^+^ cells. **(B)** Graph: Quantification of cell numbers per field with each marker from IF images in **(A)**. (n = 4). **(C–F)** Graphs: quantification of cell number from IF images with total cell number **(C)**, WT1^+^ cell number **(D)**, Ki67^+^ proliferative cell number **(E)**, and proportion of WT1^+^Ki67^+^ proliferative MPCs **(F)**. Error bars represent mean ± SD. Each plot showed different biological replicates (n = 4). **(G–I)** Graphs: RT-qPCR analysis of *WT1* mRNA expression after 3 days of culture with FGF2, PDGF-BB, SU, and CP **(G)**. *WT1* mRNA expression during long-term culture by FGF2 **(H)** and PDGF-BB treatment **(I)**. Error bars represent mean ± SD. Each plot showed different biological replicates (n = 3). Relative mRNA expression of each gene was normalized with the control basal culture condition. Scale bars = 20 μm *p < 0.05, **p < 0.01, ***p < 0.001, ****p < 0.0001, ns: no significant difference by one-way ANOVA test and *t*-test in **(C–I)**.

While the role of FGF2 and PDGF in adult mesothelial cell proliferation is known, their impact during development is little known (Mutsaers et al., 1997). To confirm each molecule’s effect on pig developing MPCs, we cultured pig MPC with FGF2 and PDGF-BB for 3 days (Figure 2). PDGF-BB was chosen as the signaling molecule for the PDGF signaling pathway due to its binding potential to all PDGF receptors (Östman, 2017). We found that FGF2 and PDGF-BB treatment increased total cell number as well as the WT1^+^ cell numbers compared to the basal condition control (Figures 2A–D). Ki67 immunostaining confirmed that FGF2 and PDGF-BB significantly increased proliferating cell numbers (Figures 2A, B, E). Notably, FGF2 and PDGF-BB induced a more than four times increase in proliferating Ki67^+^WT1^+^ MPC proportion compared with the control in the short-term culture (Figure 2F). In contrast, the treatment with SU5402, a FGFR inhibitor, and CP 673451, a PDGFR inhibitor, significantly decreased both total and WT1 cell numbers (Figures 2C, D) by inducing 30–40% of cell death, labeled by cleaved caspase 3 (CASP3) 1-day post-treatment (Supplementary Figure S3). These results suggested that the effect of endogenous FGF2 and PDGF activation cultured in the basal medium impacts ∼40% of pig MPC survival and that FGF2 and PDGF signaling may be essential for WT1^+^ MPC maintenance. To investigate the effect of FGF2 and PDGF on MPC pool expansion in the long term, we cultured the MPCs with FGF2 or PDGF-BB for 14 days and analyzed *WT1* mRNA expression by qPCR (Figures 2G–I). We found that FGF2 maintained *WT1* mRNA expression more than 5 times fold change compared to the control during long-term culture (Figure 2H), while the effect of PDGF-BB pool expansion did not significantly influence the *WT1* mRNA expression compared to the control over time (Figure 2I). These results suggest that FGF2 efficiently expands the pig MPC pools, but the PDGF-BB effect on the expansion is temporal and limited.

### 2.3 BMP4 drives differentiation of pig MPCs into SMC

**FIGURE 3:**
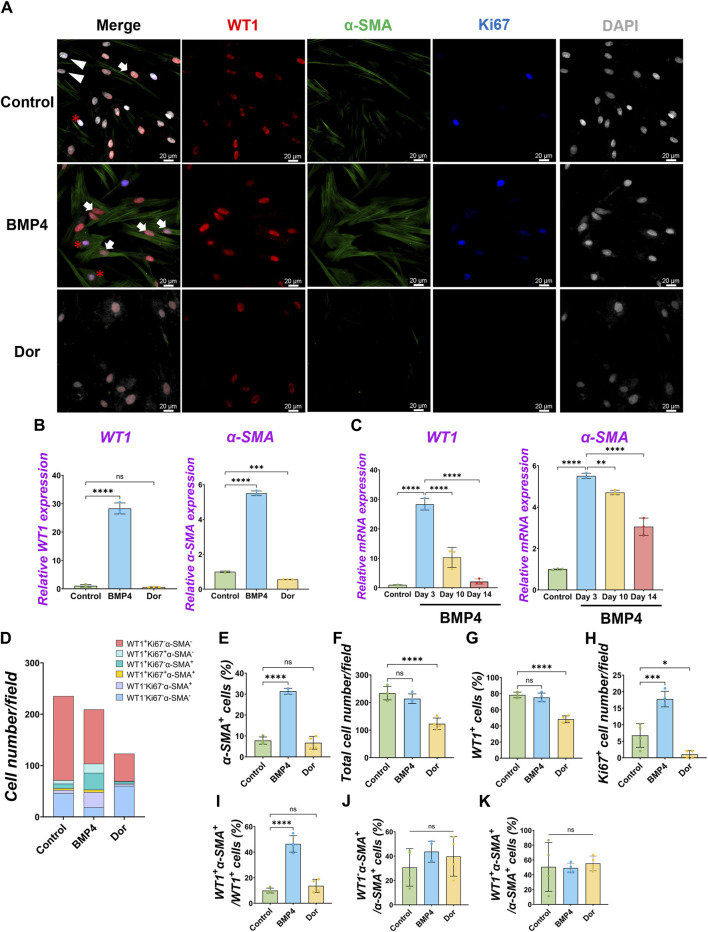
Pig parietal MPC differentiation into α-SMA^+^ smooth muscle cell by BMP4 stimulation. **(A)** Representative IF images of MPCs after 3 days of treatment with BMP4, dorsomorphin (BMP signaling inhibitor, Dor), or Control (no treatment). BMP4 induced α-SMA expression, while a Dor reduced its expression. WT1 (red), α-SMA (green), Ki67 (blue), and DAPI (grey). Arrows (white): WT1^+^α-SMA^+^ cells, asterisks: WT1^+^Ki67^+^α-SMA^+^ cells, arrowheads (white): WT1^−^α-SMA^+^ cells. **(B,C)** Graphs: RT-qPCR analysis of *WT1* and *α-SMA* mRNA expression for 3 days of MPC culture with BMP4, Dor, or Control **(B)** and long-term culture **(C)**. Error bars represent mean ± SD. Each plot showed different biological replicates (n = 3). Relative mRNA expression of each gene was normalized with the control basal culture condition. **(D)** Quantification of cell numbers per field with each marker from IF images in **(A)**. **(E–K)** Quantification of cell number from IF with α-SMA^+^ cell proportion **(E)**, total cell number **(F)**, WT1^+^ cell proportion **(G)**, Ki67^+^ proliferating cell number **(H)**, the proportion of WT1^+^α-SMA^+^ primed cells in WT1^+^ cells **(I)**, WT1^−^α-SMA^+^ cells in SMA^+^ cells **(J)**, and WT1^+^α-SMA^+^ cells in α-SMA^+^ cells **(K)**. Error bars represent mean ± SD. Each plot showed different biological replicates (n = 4). Scale bars = 20 μm *p < 0.05, **p < 0.01, ***p < 0.001, ****p < 0.0001, ns: no significant difference by one-way ANOVA test and *t*-test in **(B,C,E–K)**.

Under the pig MPC control culture condition, WT1^−^α-SMA^+^ cells were observed (5.8% ± 3.3%) (Figure 2B). We speculated that pig WT1^+^ MPCs could differentiate into smooth muscle cells (SMCs), given that mouse visceral lung mesothelial cells differentiate into smooth muscle cells during mouse lung development (De Langhe et al., 2008; Que et al., 2008). To find which signaling molecules induce pig MPC differentiation into SMC, we cultured pig MPC with various small molecules and inhibitors with different concentrations and screened *α-SMA* mRNA expression by qPCR analysis (Supplementary Figure S4A). We discovered that the BMP4 and ascorbic acid (AA) condition enhanced *α-SMA* mRNA expression compared to control among the tested conditions. Since BMP4 more dramatically induced SMC differentiation than AA, we focused on further analyses of BMP signaling. qPCR analyses found that BMP4 treatment showed significantly higher *α-SMA* mRNA induction both in short-term and long-term cultures, while BMP4 treatment had a transient effect on *WT1* mRNA increase only in the short term but did not sustain its impact in the long term (Figures 3B, C). In contrast, dorsomorphin, a BMP4 inhibitor, significantly reduced *α-SMA* mRNA expression with no significant change in *WT1* mRNA expression (Figure 3B). Since the kinetics of *WT1* and *α-SMA* mRNA by BMP4 treatment indicated the MPC differentiation into SMC, we investigated the detailed cell fate change from MPC to SMC by immunostainings in short-term culture (Figure 3A). Consistent with the qPCR observations, immunostaining analysis showed a significantly increased α-SMA^+^ cell proportion (Control: 7.8% ± 1.7% vs. BMP4: 31.4% ± 1.4%) and the number by BMP4 treatment (Figures 3A, D, E), while dorsomorphin significantly reduced the α-SMA^+^ SMC proportion (6.7% ± 3.0%). Unlike FGF2 and PDGF-BB (Figure 2), BMP4 treatment did not alter the total cell number, WT1^+^ MPC numbers, or WT1^+^ proportion but significantly increased Ki67^+^ cells (Figures 3F–H) while inducing about 20% of CASP3^+^ cell death, which might be the cell selection step (Supplementary Figure S3). Indeed, BMP4 selectively eliminates the WT1^-^Ki67^−^α-SMA^-^ unknown cell type while dorsomorphin significantly increased it (Figure 3D). Intriguingly, we observed a significantly increased proportion of WT1^+^α-SMA^+^ cells in WT1^+^ MPCs (control: 9.9% ± 1.9% vs. BMP4 group: 46.4% ± 6.4%) by BMP4 treatment (Figure 3I), but proportion of WT1^−^α-SMA^+^ in SMCs (control: 30.7% ± 15.4% vs. BMP4 group: 43.7% ± 8.6%) (Figure 3J) was not significantly changed. On the other hand, we did not observe any change in the proportion of WT1^+^α-SMA^+^ in α-SMA^+^ cells (Figure 3K). These results indicate that BMP4 treatment primes the pig mesothelial progenitor pools to co-express WT1 and α-SMA, facilitating MPC differentiation into SMCs. Based on these results, including long-term culture, we concluded that the pivotal role of BMP4 is to induce pig parietal MPC differentiation into α-SMA^+^ SMC with losing WT1 expression.

### 2.4 FGF2 and PDGF-BB suppressed pig MPC differentiation into SMCs

**FIGURE 4:**
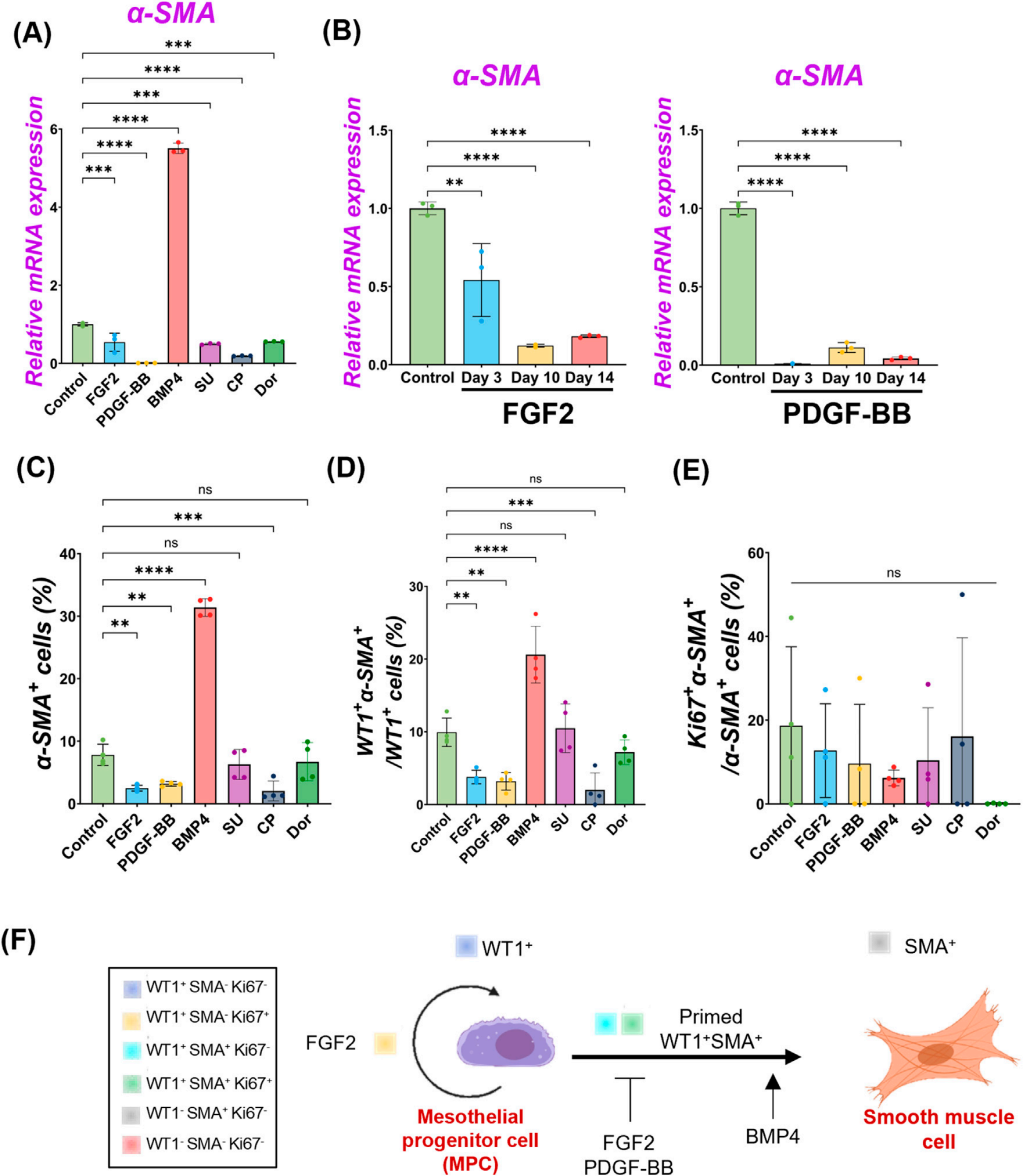
FGF2 and PDGF suppressed pig parietal MPC differentiation into smooth muscle cells. **(A,B)** Graphs: RT-qPCR analysis of α-SMA. *α-SMA* mRNA expression after 3 days of MPC culture with FGF2, PDGF-BB, BMP4, and its inhibitors (SU, CP, Dor) **(A)** and long-term culture of MPCs with FGF2, PDGF-BB **(B)**. Error bars represent mean ± SD. Each plot showed different biological replicates (n = 3). Relative mRNA expression of each gene was normalized with the control basal culture condition. **(C–E)** Graphs: Quantification of cell proportion from IF of MPCs (from Figures 2, 3) with α-SMA^+^ cell proportion **(C)**, proportion of WT1^+^α-SMA^+^ cells in WT1^+^ cells **(D)**, and proportion of Ki67^+^α-SMA^+^ cells in α-SMA^+^ cells **(E)**. Error bars represent mean ± SD. Each plot showed different biological replicates (n = 4). **(F)** Schematic summary of MPC self-renewal and differentiation into SMC by FGF2, PDGF-BB, and BMP4. **p < 0.01, ***p < 0.001, ****p < 0.0001, ns: no significant difference by one-way ANOVA test and *t*-test in **(A–E)**.

We observed pig MPC progenitor pool regulation by FGF2 and PDGF-BB (Figure 2) and differentiation into α-SMA^+^ SMC by BMP4 (Figure 3), but it was unclear whether FGF2 and PDGF-BB influence the SMC pools. To address this, we performed qPCR analyses. We found that the decreased *α-SMA* mRNA expression by the FGF2 or PDGF-BB over time (Figures 4A, B, Supplementary Figures S4), and the further analysis of IF data showed that the proportion of α-SMA^+^ cells was significantly reduced by the FGF2 or PDGF-BB treatment (Control vs. FGF2 vs. PDGF-BB groups: 7.8% ± 1.7% vs. 2.5% ± 0.5% vs. 3.2% ± 0.4%), while BMP4 significantly induced α-SMA^+^ cells (31.4% ± 1.4%) (Figure 4C). In particular, PDGF-BB showed a dramatic decrease of *α-SMA* mRNA than FGF2 (Figure 4B). While there were no significant changes in the proportion of proliferating α-SMA^+^ cells, the proportion of WT1^+^α-SMA^+^ cells was significantly decreased by the FGF2 or PDGF treatment (Control vs. FGF2 vs. PDGF-BB groups: 9.9% ± 1.9% vs. 3.8% ± 0.9% vs. 3.4% ± 1.3%) (Figures 4D, E). These results indicate that FGF2 and PDGF play a central role in pig MPC progenitor pool expansion by inhibiting the induction of WT1^+^α-SMA^+^ primed cells, leading to α-SMA^+^ smooth muscle cells (Figure 4F).

### 2.5 Dominance of FGF2 effect over BMP signaling in pig MPC pool regulation

**FIGURE 5:**
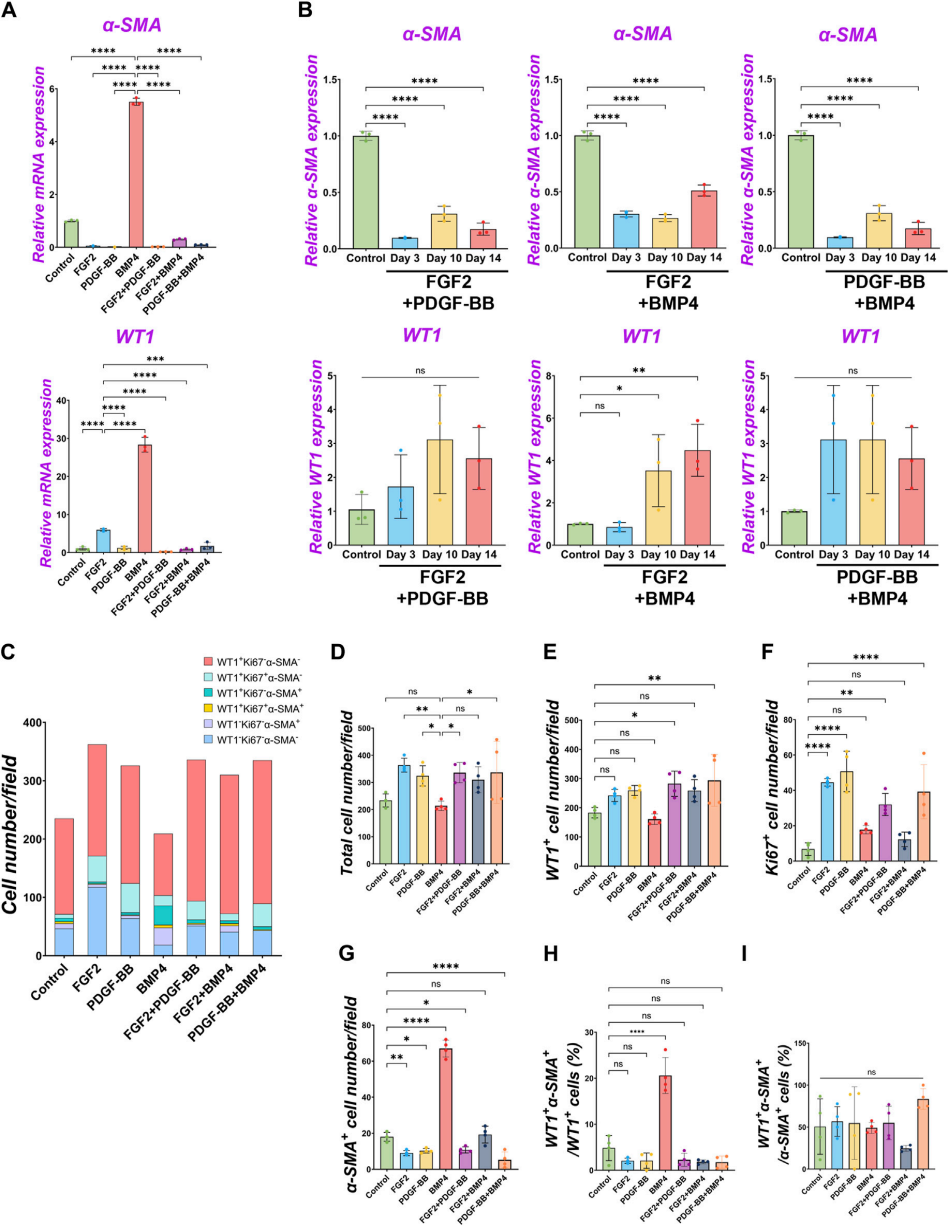
The dominance of FGF2 effect over BMP signaling in pig parietal MPC pool regulation. **(A,B)** Graphs: RT-qPCR analysis of *WT1* and *α-SMA* mRNA expression of MPC culture with signaling molecules and its combination during 3 days of culture **(A)** and long-term culture **(B)**. **(C)** Graph: Quantification of cell numbers per field with each marker from IF images. (n = 4). **(D–G)** Graphs: quantification of cell number from IF with total cell number **(D)**, WT1^+^ cells **(E)**, Ki67^+^ cells **(F)**, and α-SMA^+^ cells **(G)**. (n = 4) **(H,I)** Graphs: proportion of WT1^+^α-SMA^+^ cells in WT1^+^ cells **(H)**, proportion of WT1^+^α-SMA^+^ cells in α-SMA^+^ cells **(I)**. Error bars represent mean ± SD. Each plot showed different biological replicates (n = 4). Scale bars = 20 μm. *p < 0.05, **p < 0.01, ****p < 0.0001, ns: no significant difference by one-way ANOVA test and *t*-test in **(A,B,D–I)**.

Since we found FGF2 and PDGF suppressed BMP4-mediated MPC differentiation into SMC (Figures 2–4), we cultured pig MPCs with the combination of FGF2 and BMP4 (FGF2 + BMP4) or PDGF-BB and BMP4 (PDGF-BB + BMP4) to investigate the potential counter effect. We found that the MPC culture with FGF2 + BMP4 and PDGF-BB + BMP4 significantly suppressed the BMP4-mediated MPC differentiation into SMC with lower *α-SMA* mRNA expression than the BMP4 group (Figure 5A). This mRNA expression trend was the same in the long-term culture (Figure 5B). Although the short-term treatment with FGF2 + BMP4 and PDGF-BB + BMP4 showed a decrease in *WT1* mRNA expression (Figure 5A), the long-term effect with FGF2 + BMP4 exhibited an increase in the *WT1* mRNA expression compared to controls (Figure 5B), consistent with the FGF2 effect (Figure 2). The long-term effect of PDGF-BB + BMP4 did not impact the *WT1* mRNA expression. Interestingly, the FGF2 + BMP4 or PDGF-BB + BMP4 condition induced more cell proliferation with a higher total cell number than the BMP4 group in the short term (Figures 5C–G). In contrast, FGF2 + PDGF-BB and PDGF-BB + BMP4 conditions significantly increased WT1^+^ MPCs and proliferating cell numbers than the control condition in the short-term but could not sustain *WT1* mRNA expression in the long-term (Figures 5A, E, F). FGF2 + PDGF-BB and PDGF-BB + BMP4 conditions significantly decreased α-SMA^+^ cells and showed no increase of primed WT1^+^α-SMA^+^ cells in WT1^+^ cells (Figures 5G, H). As we expected, there was no significant change in WT1^+^α-SMA^+^ cells in α-SMA^+^ cells (Figure 5I). These results suggest the critical role of FGF2 in maintaining the MPC pool and its self-renewal that counteracts the BMP signaling effects on pig MPC differentiation into SMC.

### 2.6 Wnt signaling facilitates pig MPC maturation

During development, mesenchymal β-catenin signaling controls parabronchial smooth muscle cell (PSMC) progenitors in the sub-mesothelial mesenchyme (De Langhe et al., 2008). Wnt signaling is involved in the outer mesothelial pool size of the zebrafish swimbladder during development (Davidenko et al., 2016). However, the molecular characterization of pig MPCs and their maturation during pig lung development have been little studied. To address this issue, we performed immunostaining of WT1 and CALB2 in pig and mouse lung development (Supplementary Figure S5). Developing porcine pleural mesothelial cells expressed high levels of WT1 in the E26 early pseudoglandular stage of porcine lungs, but the relative expression level in the peripheral layer of the lungs was decreased to less than 1/4 of signal intensity in the later stage (Supplementary Figure S5A). In contrast, CALB2 expression was not detected in the peripheral layer of the pig lungs in the E26 and E40 early pseudoglandular stage but appeared in the canalicular stage and afterward (Supplementary Figure S5D). These results indicate that CALB2 is the marker for mesothelial cell maturation during porcine lung development. Intriguingly, CALB2-labeled non-peripheral cells were also observed around the sub-pleural region of the porcine lungs on days 90 and 104 (Supplementary Figure S5D), reminiscent of mouse lung mesothelial cell migration inside of the developing lungs using WT1 lineage tracing analysis (Dixit et al., 2013). We also confirmed that the WT1 expression pattern was also similar during mouse lung development, supported by previous studies (Que et al., 2008; Dixit et al., 2013) (Supplementary Figure S5C), while CALB2 started to be expressed in the sub-peripheral layer from the E14.5 pseudoglandular stage in mouse lung development (Supplementary Figure S5F).

To investigate the common MPC maturation markers across the species, we revisited the deposit single-cell RNA-seq (scRNA-seq) database of developing human (He et al., 2022) and mouse (Negretti et al., 2021) lung mesenchyme (Supplementary Figure S6). We found that *WT1* was highly expressed in the early pseudoglandular stage but decreased its expression in the late pseudoglandular and canalicular stages of human and mouse-developing lungs. *CALB2*, a mature mesothelial cell marker, was slightly observed but not abundant in human lung development. During mouse lung development, *CALB2* was observed in non-mesothelial cells. In contrast, mesothelin (*MSLN*) expression was observed in the late pseudoglandular stage of developing human lungs to the canalicular stage while around the E18 sacculation stage and afterward in the mouse lungs. These results suggest that decreased expression of *WT1* and increased *MSLN* are the evolutionarily conserved markers for MPC maturation, but *CALB2* is a pig-specific unique marker for MPC maturation. Based on these results, we examined pig MPC maturation in an *in vitro* study using *WT1*, *CALB2*, and *MSLN*.

**FIGURE 6:**
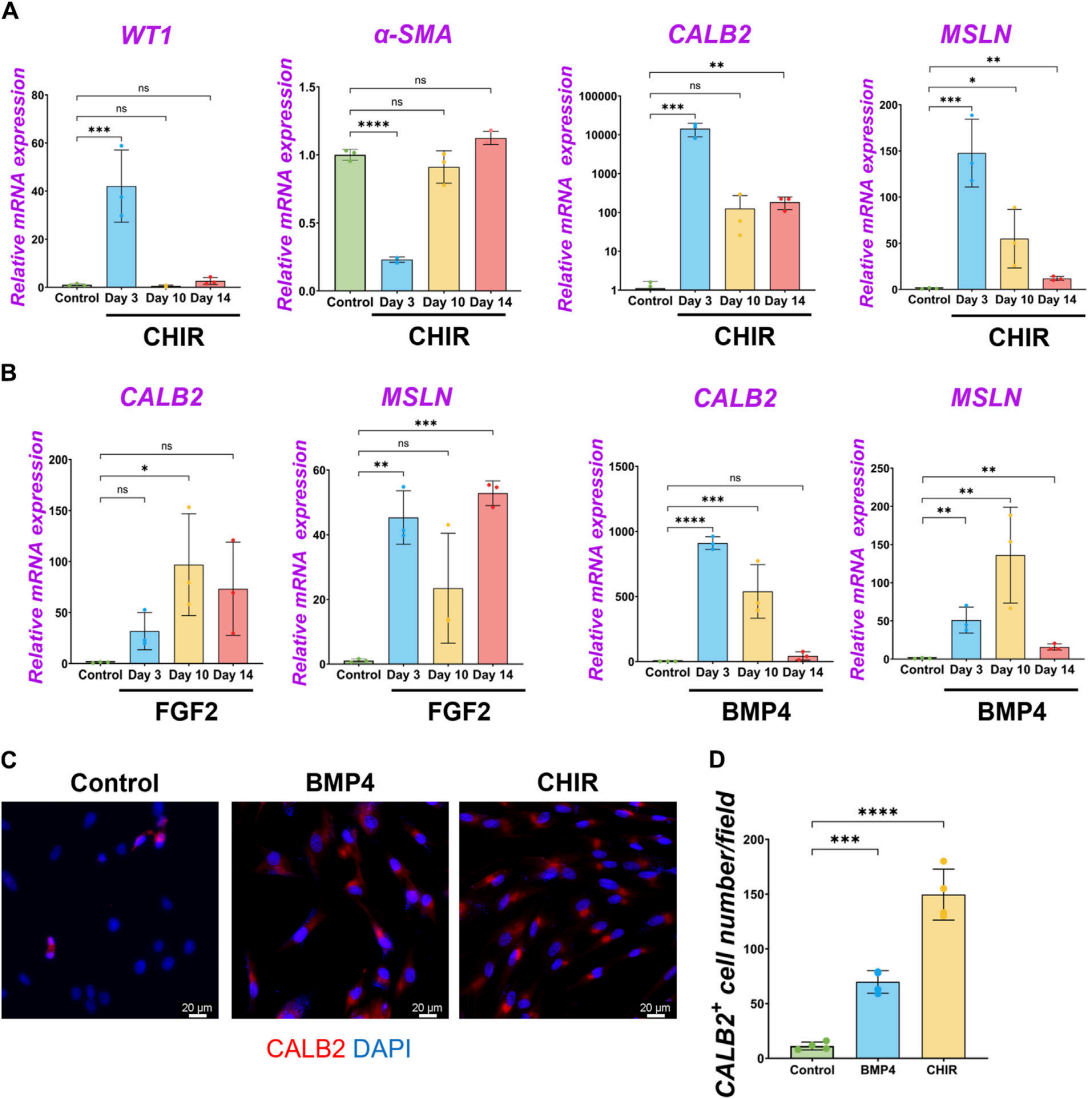
β-catenin (wnt) activation induced the maturation of pig parietal MPCs to CALB2^+^ mature mesothelial cells. **(A,B)** Graphs: RT-qPCR analysis of *WT1*, *α-SMA*, *CALB2*, and *MSLN* mRNA expression for long-term culture of MPCs with CHIR99021 (CHIR) **(A)**, and FGF2, BMP4 **(B)**. Error bars represent mean ± SD. Each plot showed different biological replicates (n = 3). Relative mRNA expression of each gene was normalized with the control basal culture condition. **(C)** Representative IF images of MPCs after 3 days of treatment with BMP4 and CHIR. CALB2 (red), DAPI (blue). **(D)** Graph: quantification of CALB2^+^ cell number from IF. Error bars represent mean ± SD. Each plot showed different biological replicates (n = 4). Scale bars = 20 μm. *p < 0.05, **p < 0.01, ***p < 0.001, ****p < 0.0001, ns: no significant difference by one-way ANOVA test and *t*-test in **(A,B,D)**.

We performed qPCR to screen the most potent signaling molecules regulating pig MPC maturation to CALB2^+^ and MSLN^+^ mature mesothelial cells (Supplementary Figure S4B). Among them, we found that most signaling molecules induced the upregulation of *CALB2* and *MSLN* mRNA. In particular, the GSK3β inhibitor that acts as a Wnt activator (CHIR) showed the most dramatic increase in *CALB2* mRNA expression. Thus, we focused on analyzing Wnt signaling using CHIR in the pig MPC maturation. Three days of short-term CHIR treatment increased *WT1*, *CALB2*, and *MSLN* mRNA expressions, while the long-term CHIR treatment lost WT1^+^ MPC pools but relatively sustained *CALB2* expression (Figure 6A). Since high *WT1* mRNA expression is the landmark for immature MPC pool expansion, these results indicate that the pig MPC maturation by CHIR occurred as a long-term effect (Figure 6A). Interestingly, we also found that long-term treatment with FGF2 or BMP4 significantly increased *MSLN* mRNA expression compared to the control (Figure 6B). However, FGF2 did not increase the mRNA expression of *MSLN* and *CALB2* in a dose-dependent manner in short-term culture, while BMP4 induced *CALB2* mRNA expression in a dose-dependent manner (Supplementary Figure S4C). Furthermore, the *CALB2* mRNA upregulation by FGF2 or BMP4 was transient and relatively limited in the long-term treatment compared to the CHIR treatment (Figure 6B). Consistent with the qPCR results, the CALB2 immunostaining exhibited a consistent trend with qPCR results, indicating the increased CALB2^+^ cells by CHIR treatment (Figures 6C, D). As shown in the PDGF-BB effect, CHIR induced Ki67^+^ proliferative WT1^+^ cells and significantly increased total cell number compared to control (Supplementary Figure S7A), while no WT1^+^ cell number or proportional change and reduced α-SMA^+^ cell number (Supplementary Figure S7D). These results indicate that Wnt signaling activation induces pig MPC maturation into MSLN^+^ CALB2^+^ cells, corresponding to the expression pattern of CALB2 in porcine lung development.

## 3 Discussion

Previous studies showed the markers of adult mesothelial cells or in mesothelioma, but it has been unclear how developing mesothelial progenitors shift the marker expressions and their association with cellular behaviors. We established pig and mouse MPC expansion protocols that allow us to find the foundation of signaling pathways involved in MPC pool expansion, differentiation, and maturation. Technically, we could not expand the cells from the E40 or earlier time point’s thoracic wall in either method due to the low effectiveness of isolating MPCs even using swine specimens larger than mice (data not shown). Harvesting MPCs exclusively from the lungs was also challenging because it contained various other cell types after the culture (data not shown). Based on these technical limitations, we focused on the MPC cellular analysis derived from the E80 thoracic walls. Of note, we also expand mouse MPCs, in this culture condition, from the thorax at E17.0 ∼ E17.5 canalicular ∼ sacculation stage, corresponding to E80 pig developmental time point. The WT1 lineage-tracing mouse model showed that less than 1% of WT1^+^ cells were identified after sorting by FACS, and thereby, it was challenging to expand them efficiently post-sorting (Supplementary Figure S1G). Further optimization using these mice and sorting methods combined with our established isolation method is required.

FGF signaling pathways have been classically known as critical mitogens for both epithelium and mesenchyme (Lebeche et al., 1999; Ornitz and Itoh, 2001; Yuan et al., 2018). Interestingly, mesothelial cells and mesothelioma have been characterized as epithelial-like and mesenchymal-like features (Travis et al., 2004; Koopmans and Rinkevich, 2018). We found that FGF2 has the most potent effect on MPC self-renewal in the long-term culture among tested conditions and inhibits BMP4-mediated SMC differentiation. Given that FGF2 high expression in mesothelioma is one of the critical prognosis factors and carcinogenesis often renders developmental program (Perantoni et al., 1995; Dudley et al., 1999; Schelch et al., 2018), we speculate that targeting therapy for the FGF2 and its downstream, such as Spry2 (García-Domínguez et al., 2011), Ras (Ichise et al., 2014), or Sos (Tan et al., 2020), may be critical for controlling FGF2^high+^ mesothelioma expansion and metastasis.

We found BMP4 signaling was critical for inducing MPC differentiation into SMC with an increase of α-SMA^+^ cells, including primed, transitioning WT1^+^α-SMA^+^ cells and differentiated WT1^−^α-SMA^+^ cells (Figure 4). The molecular mechanism of how BMP4 converts MPC to SMC needs to be determined in the future. Interestingly, our immunostaining analyses revealed that proliferating Ki67^+^α-SMA^+^ cells were never observed without tuning on WT1 (Figure 4). BMP4 initially induced WT1^+^Ki67^+^α-SMA^+^ transitioning cells but later lost the *WT1* mRNA expression (Figure 3B), suggesting that the critical role of BMP4 in MPC cell fate change to post-mitotic terminally differentiated SMCs. Since retinoic acid treatment for acute leukemia patients induces terminally differentiated cells and is an effective therapy for those patients (Stahl and Tallman, 2019), how BMP4 signaling activation would influence mesothelioma would be an attractive question.

Parietal MPCs and lung peripheral MPCs showed distinct morphology and function (Shelton et al., 2013). Our study showed that potential CALB2 descendants of MPCs appeared around the neighboring WT1^+^ mesothelium (Supplementary Figure S5D), supported by previous studies of mouse lung development (Blum et al., 2015). There are remaining exciting questions regarding MPC maturation: about the role of CALB2 in porcine parietal MPC, its developmental distributions, how the parietal and lung-peripheral MPC distinctively mature, and how these MPC pools communicate during development. Intriguingly, WT1 is a known prognostic factor for mesothelioma, but CALB2 is not (Cedrés et al., 2014). Since we observed higher expression of WT1 in immature mesothelial cells and lower expression in mature cells, and the reciprocal CALB2 expression pattern, the malignancy of mesothelioma is potentially related to the self-renewal capacity or immaturity of mesothelial cells regardless of its maturation or the defects of its maturation.

Interestingly, we did not observe CALB2^+^ cells on the parietal mesothelium during mouse development (Supplementary Figure S5F). We examined three different antibodies against MSLN to investigate the maturation of MPC during development. However, MSLN expression was not detected in developing lungs and thorax, as in the previous study (Dixit et al., 2013), which is inconsistent with the scRNA-seq result (Supplementary Figure S6B). This indicates that protein expression may be regulated at post-translational levels or require further technical advancements.

Interestingly, the WT1^+^ MPCs showed α-SMA expression, reminiscent of porcine parietal mesothelial cells in the E26 early pseudoglandular stage (Figure 1E; Supplementary Figure S5A), while it is uncommon in peripheral lung MPCs. In our culture model, we used MPCs at the canalicular ∼ sacculation stage. Our results indicate that porcine parietal MPCs may be a source of SMCs around the developing ribs.

**FIGURE 7:**
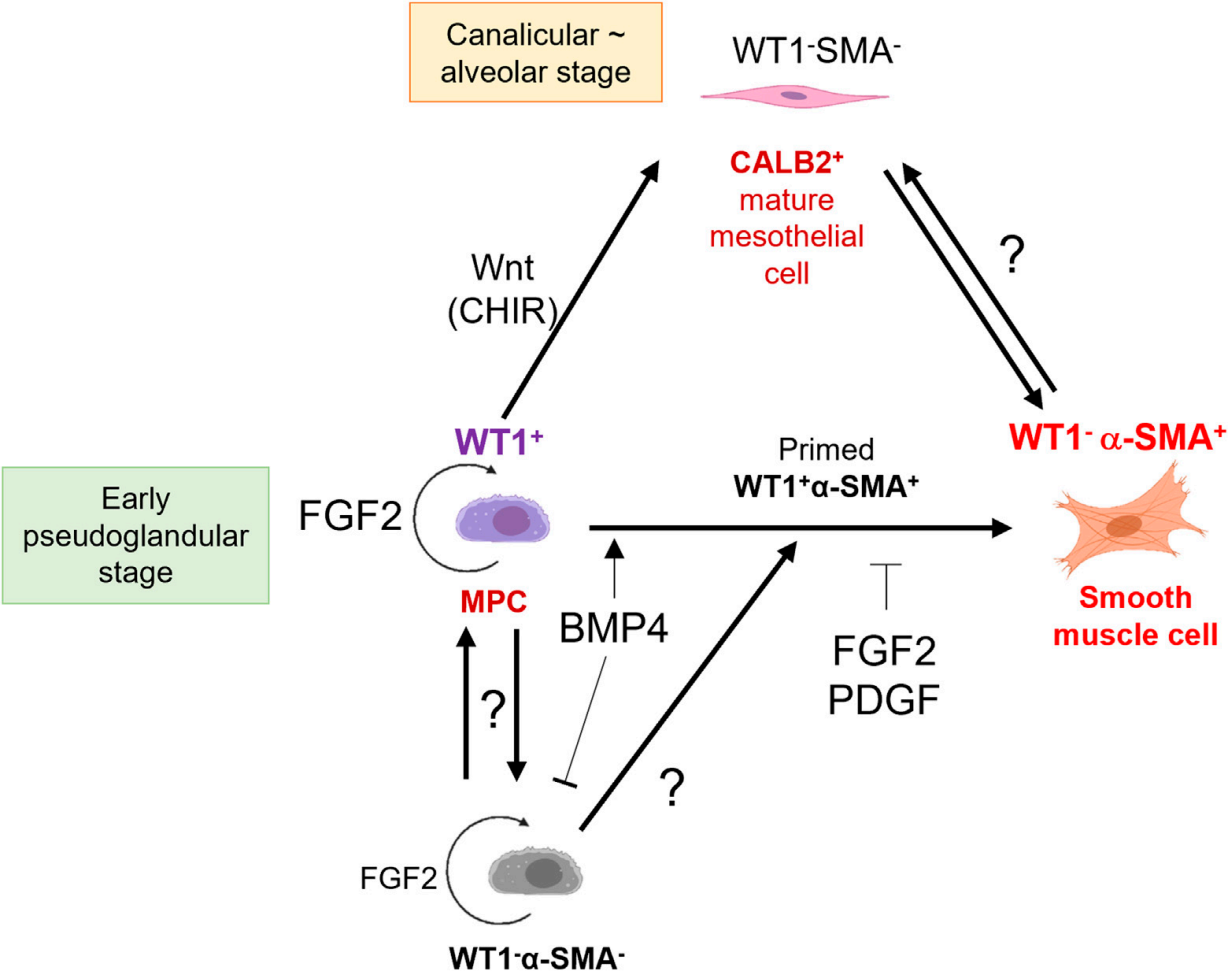
Schematic model of embryonic pig parietal MPC behavior control by intertwined signaling. FGF2 induces self-renewal of WT1^+^ MPCs. MPCs differentiate into α-SMA^+^ SMCs through primed WT1^+^α-SMA^+^ cells by BMP4 stimulation. FGF and PDGF signaling suppresses the BMP4-mediated SMC differentiation. Developing mesothelium shows stage-specific markers: high WT1 expression in the early pseudoglandular stage of porcine lung development and low WT1 expression and CALB2 expression in the calanlicular ∼ alveolar stage. Wnt activation by CHIR facilitates the MPC maturation process. The role of unknown WT1^−^α-SMA^-^ pools in MPC proliferation and differentiation is unclear.

We summarized MPC fate change by signaling molecules (Figure 7). Interestingly, FGF2 promoted the expansion of both WT1^+^ MPCs and WT1^−^α-SMA^-^ pool compared to the control (Figure 2B). The WT1^−^α-SMA^-^ pool would involve CALB2^+^ mature mesothelial cells. However, BMP4 suppressed the WT1^−^α-SMA^-^ pool expansion (Figure 3D), while BMP4 also increased CALB2 expression in short-term culture (Figures 6B, D). This discrepancy suggests the existence of unknown WT1^−^α-SMA^-^CALB2^-^ pool, which may have a role in the MPC regulation (Figure 7). We also observed the expansion of WT1^+^ cells, which correlates to the expansion of WT1^-^ cells when we added FGF2, as shown in Figure 2B. Based on this, the origin of WT1^+^ cells can be derived from the WT1^-^ cells, potentially neighboring thorax mesenchymal cells. The role of WT1 in parietal MPCs is also not investigated yet. Further analysis using genetic lineage tracing or single cell level bioinformatics analysis may reveal the lineage hierarchy, parietal MPC vs. peripheral lung MPC vs. WT1^−^α-SMA^-^ niche interactions, and association with mesothelioma, which will lead to further understanding of mesothelial development and pathogenesis.

## 4 Methods

### 4.1 Animals

All surgical procedures were conducted under the approval of the Columbia University Institutional Animal Care and Use Committee and USAMRMC Animal Care and Use Review Office (ACURO). For pig experiment, Timed-pregnant Yucatan miniature sows were obtained from Sinclair BioResources. For mouse experiment, CD-1 mice (male, 8 weeks), (female, 8 weeks) were purchased from Charles River Laboratories. For WT1 lineage-tracing mouse analysis, WT1^tm2(cre/ERT2)Wtp^ mouse (male, 8 weeks), Gt (ROSA)26Sor^tm14(CAG-tdTomato)Hze^ (female, 8 weeks) were purchased from Jackson Laboratory (Farmington, CT).

### 4.2 Parietal pig mesothelial progenitor cell (MPC) isolation

E80 Yucatan pig embryos were surgically harvested from Yucatan pig sows. After euthanasia, the thorax was harvested. Two methods for MPC isolation were compared as shown in Figure 1: 1) Mesothelial tissue was isolated from the medial side of the chest wall of E80 pigs with a cell scraper (Fisher Scientific, Waltham, MA) and incubated in a 50 mL tube containing 2 mL of 0.05% or 0.25% trypsin-EDTA (Thermofisher Scientific, Waltham, MA) solution at 37°C for 20 min 2) 0.05% or 0.25% trypsin-EDTA were directly treated on the thoracic wall, followed by incubation for at 37°C for 20 min. After trypsin-EDTA treatment in either method, the dissociated cell pellet was washed with PBS × 3 times by centrifugation (350 × g for 5 min) at 4°C. The cell pellet was then incubated with Red Blood Cell (RBC) lysis buffer (Biolegend, San Diego, CA) at 4°C for 5 min. After centrifugation (350 × g for 5 min, at 4°C), the supernatant was aspirated and the cell pellet was three times with PBS. After washing with PBS, the cell pellet was filtered through a cell strainer (pore size 40 um, MTC Bio) and seeded onto type I collagen (10 ug/cm^2^, rat tail derived, Sigma-Aldrich, Burlington, MA)-coated six well tissue culture plates. MPCs (P0) were seeded in MPC culture medium (DMEM (high glucose, Gibco) + 10% FBS (Clytia, Marlborough, MA) + 1% pen/strep (Gibco)) for 7 days. During culture, the MPC culture medium was replaced every other day. For passage, MPCs were washed with PBS and dissociated in 0.05% trypsin-EDTA for 5 min at 37°C. For MPC culture and analysis, passage 6-8 MPCs grown on gelatin-coated tissue culture plates were used.

### 4.3 Mouse mesothelial progenitor cell (MPC) isolation

Mouse parietal MPCs were isolated from the thorax of E17.5 embryos with the pre-treatment of 0.5 mL of 0.05% or 0.25% trypsin-EDTA onto the median side of the thorax. Then, the parietal mouse MPCs were treated as pig MPC isolation. The mouse MPC was cultured on a type I collagen (10 ug/cm^2^)-coated 24well tissue culture plate in MPC culture medium with the replacement of the cell culture media every other day. Mouse MPCs isolated from E17.5 WT1 lineage-tracing mouse embryo were conducted with the same way. For quantification of tdTomato^+^ mouse parietal MPCs isolated from E17.5 WT1 lineage-tracing mouse (3 mg/kg tamoxifen administration into pregnant mother at E15.5, E16.5 by oral gavage feeding) by flow cytometry. The isolated mouse MPCs were stained with DAPI (Cayman Chemical, Ann Arbor, MI), rat to mouse EpCAM antibody (BV711-conjugated, Biolegend), rat to mouse PECAM antibody (APC-conjugated, Biolegend), CD45 (BV605-conjugated, BD Biosciences, San Jose, CA), and subsequently analyzed by flow cytometry (Sony MA900, Sony Biotechnology, San Jose, CA). The sorted CD45^−^ EpCAM^−^ PECAM^−^ tdTomato^+^ fractions were cultured on a type I collagen-coated 24 well tissue culture plate. After 3 days of culture, the cell nucleus was stained with WT1 and NucBlue™ Live ReadyProbes™ Reagent (Fisher Scientific). More detailed information of antibodies were described in Supplementary Table S2. The stained cells were visualized with a Leica DMI microscope (Leica, New York, NY).

### 4.4 Parietal pig mesothelial progenitor cell (MPC) culture

To investigate the MPC cell fate by signaling molecules, MPCs were cultured in the MPC culture medium with various signaling molecules (FGF2 (2/10/20 ng/mL) (Peprotech, Cranbury, NJ), PDGF-BB (1/5/15 ng/mL) (R&D systems, Minneapolis, MN), BMP4 (5/25/50 ng/mL) (R&D systems), retinoic acid (0.1/0.5/1 uM) (RA, Sigma-Aldrich), CHIR99021 (0.3/1.5/3 uM) (MedChem Express, Hoboken, NJ), ascorbic acid (5/25/50 ug/mL) (AA, Thermofisher Scientific), purmorphamine (0.2/1/2uM) (sonic hedgehog (Shh) activator, Tocris, Westwoods Bus Park Ellisville, MO)) and the inhibitors (SU5402 as FGFR inhibitor (30 nM) (MedChem Express), CP673451 as PDGFR inhibitor (4 nM) (MedChem Express), and dorsomorphin (2 uM) (Tocris) for 3, 10, or 14 days. During MPC culture with signaling molecules for immunofluorescence (IF) and qPCR analysis, 20 ng/mL of FGF2, 15 ng/mL of PDGF-BB, 50 ng/mL of BMP4, and three uM of CHIR 99021 were used. The MPC culture medium, including signaling molecules, was replaced every other day and the MPCs were passaged at day 3, 6, and 10 to avoid full confluency.

### 4.5 RT-qPCR

mRNA was isolated from MPCs with Direct-zol RNA Microprep isolation kit (Zymo Research, Irvine, CA) after lysis of MPCs with IBI isolate total reagent (IBI Scientific, Dubuque, IA). For cDNA synthesis, the isolated mRNA was mixed with PrimeScript RT Master Mix (Takara bio, San Jose, CA), followed by cDNA synthesis protocol. For RT-qPCR analysis, the synthesized cDNA was mixed with qPCR primers (see each primer sequence in Supplementary Table S1) and Luna universal qPCR Master Mix (New England Biolabs (NEB), Ipswich, MA). RT-qPCR was conducted with Quantstudio (Thermofisher Scientific). mRNA expression of each gene was normalized with the housekeeping gene (GAPDH). The relative mRNA expression of the genes was normalized with the control group (MPC culture in DMEM +10% FBS +1% pen/strep).

### 4.6 Immunofluorescence (IF)

For cell sample preparation, MPCs were fixed with 3.7% paraformaldehyde (PFA) for 10 min at room temperature. For tissue sample preparation, 10um-frozen sectioned tissue samples were washed with PBS 3 times, followed by antigen retrieval with citrate-based buffer (Vector Laboratories) in the microwave for 8 min. After washing the cells and the tissue samples with PBS 3 times, the primary antibodies in dilution solution (0.25% triton X-100 (Sigma-Aldrich) + 0.75% BSA in PBS) were treated to the samples and incubated at 4°C for overnight. After 3 times PBS wash on the following day, the secondary antibodies and DAPI were treated (0.75% BSA in PBS) for 1 h at room temperature. Then, the sample was mounted with a coverglass, anti-fade reagent (Invitrogen). For pig cell/tissue CALB2 staining, primary antibody-treated samples were treated with HRP conjugated anti-chicken antibody (in PBS) and incubated for 30 min at room temperature. After PBS wash, Cy3 tyramide (AAT Bioquest, Pleasanton, CA) (1:1000 diluted in 100 mM borate +0.1% Tween-20 + 0.003% H_2_O_2_ solution (pH 8.5)) was treated in the samples and incubated for 15 min at room temperature in the dark. After PBS wash, the samples were mounted with a coverglass and an anti-fade reagent (Invitrogen). The cell samples were visualized with a Leica DMI microscope. The tissue samples were visualized with a Zeiss confocal microscope (Zeiss, White Plains, NY). More detailed information of the used antibodies in this study was described in Supplementary Table S2.

### 4.7 RNA-seq data analysis

For human and mouse RNA-seq data analysis, we utilized the database from the previous studies. The hyperlink of the database is described in Data Availability Statement.

### 4.8 Statistical analysis

Quantification of cell number in the phase contrast images was conducted by ImageJ. For immunostained cell (single-immunostained and co-immunostained cell population) and DAPI-stained cell counting from IF images, Cellpose software was used. The mean fluorescence intensity (MFI) of each IF sample was measured in the non-overlapping random fields using ImageJ software. Data analysis was performed using Prism 10. Data acquired by performing biological replicates ((n = 3) for RT-qPCR and phase contrast images, (n = 4) for IF images) of three or four independent experiments are presented as the mean ± standard deviation (SD). Statistical significance was determined using a one-way ANOVA or a two-tailed *t*-test. *p < 0.05, **p < 0.01, ***p < 0.001, ****p < 0.0001, ns: non-significant.

## Data Availability

The datasets presented in this study can be found in online repositories. The names of the repository/repositories and accession number(s) can be found in the article/Supplementary Material.

## References

[B1] Blum W. Pecze L. Felley-Bosco E. Schwaller B. (2015). Overexpression or absence of calretinin in mouse primary mesothelial cells inversely affects proliferation and cell migration. Respir. Res. 16 (1), 153. 10.1186/s12931-015-0311-6 26695618 PMC4699379

[B2] Boutin C. Schlesser M. Frenay C. Astoul P. (1998). Malignant pleural mesothelioma. Eur. Respir. J. 12 (4), 972–981. 10.1183/09031936.98.12040972 9817178

[B3] Breborowicz A. Korybalska K. Grzybowski A. Wieczorowska-Tobis K. Martis L. Oreopoulos D. G. (1996). Synthesis of hyaluronic acid by human peritoneal mesothelial cells: effect of cytokines and dialysate. Perit. Dial. Int. 16 (4), 374–378. 10.1177/089686089601600410 8863330

[B4] Cano E. Carmona R. Muñoz-Chápuli R. (2013). Wt1-expressing progenitors contribute to multiple tissues in the developing lung. Am. J. Physiology - Lung Cell. Mol. Physiology 305 (4), L322–L332. 10.1152/ajplung.00424.2012 23812634

[B5] Cedrés S. Montero M. A. Zamora E. Martínez A. Martínez P. Fariñas L. (2014). Expression of Wilms’ tumor gene (WT1) is associated with survival in malignant pleural mesothelioma. Clin. Transl. Oncol. 16 (9), 776–782. 10.1007/s12094-013-1146-6 24323466

[B6] Davidenko N. Schuster C. F. Bax D. V. Farndale R. W. Hamaia S. Best S. M. (2016). Evaluation of cell binding to collagen and gelatin: a study of the effect of 2D and 3D architecture and surface chemistry. J. Mater. Sci. Mater. Med. 27 (10), 148. 10.1007/s10856-016-5763-9 27582068 PMC5007264

[B7] De Langhe S. P. Carraro G. Tefft D. Li C. Xu X. Chai Y. (2008). Formation and differentiation of multiple mesenchymal lineages during lung development is regulated by beta-catenin signaling. PLoS ONE 3 (1), e1516. 10.1371/journal.pone.0001516 18231602 PMC2211394

[B8] Dixit R. Ai X. Fine A. (2013). Derivation of lung mesenchymal lineages from the fetal mesothelium requires hedgehog signaling for mesothelial cell entry. Dev. Camb. Engl. 140 (21), 4398–4406. 10.1242/dev.098079 PMC4007715 24130328

[B9] Dudley A. T. Godin R. E. Robertson E. J. (1999). Interaction between FGF and BMP signaling pathways regulates development of metanephric mesenchyme. Genes Dev. 13 (12), 1601–1613. 10.1101/gad.13.12.1601 10385628 PMC316794

[B10] García-Domínguez C. A. Martínez N. Gragera T. Pérez-Rodríguez A. Retana D. León G. (2011). Sprouty2 and spred1-2 proteins inhibit the activation of the ERK pathway elicited by cyclopentenone prostanoids. PLoS ONE 6 (2), e16787. 10.1371/journal.pone.0016787 21364986 PMC3043057

[B11] Gilbert R. M. Schappell L. E. Gleghorn J. P. (2021). Defective mesothelium and limited physical space are drivers of dysregulated lung development in a genetic model of congenital diaphragmatic hernia. Dev. Camb. 148 (10), dev199460. 10.1242/DEV.199460 PMC8180258 34015093

[B12] Gueugnon F. Leclercq S. Blanquart C. Sagan C. Cellerin L. Padieu M. (2011). Identification of novel markers for the diagnosis of malignant pleural mesothelioma. Am. J. Pathology 178 (3), 1033–1042. 10.1016/j.ajpath.2010.12.014 PMC3070574 21356356

[B13] He P. Lim K. Sun D. Pett J. P. Jeng Q. Polanski K. (2022). A human fetal lung cell atlas uncovers proximal-distal gradients of differentiation and key regulators of epithelial fates. Cell 185 (25), 4841–4860.e25. 10.1016/J.CELL.2022.11.005 36493756 PMC7618435

[B14] Huang J. Chan S. C. Pang W. S. Chow S. H. Lok V. Zhang L. (2023). Global incidence, risk factors, and temporal trends of mesothelioma: a population-based study. J. Thorac. Oncol. 18 (6), 792–802. 10.1016/j.jtho.2023.01.095 36775192

[B15] Ichise T. Yoshida N. Ichise H. (2014). FGF2-induced Ras-MAPK signalling maintains lymphatic endothelial cell identity by upregulating endothelial-cell-specific gene expression and suppressing TGFβ signalling through Smad2. J. Cell Sci. 127 (4), 845–857. 10.1242/jcs.137836 24357720

[B16] Kawai N. Ouji Y. Sakagami M. Tojo T. Sawabata N. Yoshikawa M. (2019). Isolation and culture of pleural mesothelial cells. Exp. Lung Res. 45 (5–6), 151–156. 10.1080/01902148.2018.1511002 31250673

[B17] Kienzle A. Servais A. B. Ysasi A. B. Gibney B. C. Valenzuela C. D. Wagner W. L. (2018). Free-floating mesothelial cells in pleural fluid after lung surgery. Front. Med. 5 (APR), 89. 10.3389/fmed.2018.00089 PMC5895720 29675416

[B18] Koopmans T. Rinkevich Y. (2018). Mesothelial to mesenchyme transition as a major developmental and pathological player in trunk organs and their cavities. Commun. Biol. 1 (Issue 1), 170. 10.1038/s42003-018-0180-x 30345394 PMC6191446

[B19] Kumar-Singh S. Weyler J. Martin M. J. H. Vermeulen P. B. Van Marck E. (1999). Angiogenic cytokines in mesothelioma: a study of VEGF, FGF-1 and -2, and TGF beta expression. J. Pathology 189 (1), 72–78. 10.1002/(SICI)1096-9896(199909)189:1<72::AID-PATH401>3.0.CO;2-0 10451491

[B20] Lebeche D. Malpel S. Cardoso W. V. (1999). Fibroblast growth factor interactions in the developing lung. Mech. Dev. 86 (1–2), 125–136. 10.1016/s0925-4773(99)00124-0 10446271

[B21] Manzo G. (2019). Similarities between embryo development and cancer process suggest new strategies for research and therapy of tumors: a new point of view. Front. Cell Dev. Biol. 7 (MAR), 20. 10.3389/fcell.2019.00020 30899759 PMC6416183

[B22] McGeady T. A. Quinn P. J. Fitzpatrick E. S. Ryan M. T. Kilroy D. Lonergan P. (2017). “Veterinary embryology,” in Veterinary anatomy and physiology 2nd edition (Wiley-Blackwell), 400. Available at: https://www.wiley.com/en-us/Veterinary+Embryology,+2nd+Edition-p-9781118940617.

[B23] Mierzejewski M. Paplinska-Goryca M. Korczynski P. Krenke R. (2021). Primary human mesothelial cell culture in the evaluation of the inflammatory response to different sclerosing agents used for pleurodesis. Physiol. Rep. 9 (8), e14846. 10.14814/phy2.14846 33932124 PMC8087983

[B24] Miura A. Sarmah H. Tanaka J. Hwang Y. Sawada A. Shimamura Y. (2023). Conditional blastocyst complementation of a defective Foxa2 lineage efficiently promotes the generation of the whole lung. ELife 12, e86105. 10.7554/eLife.86105 37861292 PMC10642968

[B25] Mutsaers S. E. McAnulty R. J. Laurent G. J. Versnel M. A. Whitaker D. Papadimitriou J. M. (1997). Cytokine regulation of mesothelial cell proliferation *in vitro* and *in vivo* . Eur. J. Cell Biol. 72 (1), 24–29. 9013722

[B26] Namvar S. Woolf A. S. Zeef L. A. H. Wilm T. Wilm B. Herrick S. E. (2018). Functional molecules in mesothelial-to-mesenchymal transition revealed by transcriptome analyses. J. Pathology 245 (4), 491–501. 10.1002/path.5101 PMC6055603 29774544

[B27] Negretti N. M. Plosa E. J. Benjamin J. T. Schuler B. A. Habermann A. C. Jetter C. S. (2021). A single-cell atlas of mouse lung development. Dev. Camb. Engl. 148 (24), dev199512. 10.1242/dev.199512 PMC8722390 34927678

[B28] Obacz J. Yung H. Shamseddin M. Linnane E. Liu X. Azad A. A. (2021). Biological basis for novel mesothelioma therapies. Br. J. Cancer 125 (Issue 8), 1039–1055. 10.1038/s41416-021-01462-2 34226685 PMC8505556

[B29] Ornitz D. M. Itoh N. (2001). Fibroblast growth factors. Genome Biol. 2 (Issue 3), REVIEWS3005. 10.1186/gb-2001-2-3-reviews3005 11276432 PMC138918

[B30] Östman A. (2017). PDGF receptors in tumor stroma: biological effects and associations with prognosis and response to treatment. Adv. Drug Deliv. Rev. 121, 117–123. 10.1016/j.addr.2017.09.022 28970051

[B31] Perantoni A. O. Dove L. F. Karavanova I. (1995). Basic fibroblast growth factor can mediate the early inductive events in renal development. Proc. Natl. Acad. Sci. U. S. A. 92 (10), 4696–4700. 10.1073/pnas.92.10.4696 7753867 PMC42011

[B32] Pruett N. Singh A. Shankar A. Schrump D. S. Hoang C. D. (2020). Normal mesothelial cell lines newly derived from human pleural biopsy explants. Am. J. Physiology - Lung Cell. Mol. Physiology 319 (4), L652–L660. 10.1152/AJPLUNG.00141.2020 PMC7642897 32726133

[B33] Que J. Wilm B. Hasegawa H. Wang F. Bader D. Hogan B. L. M. (2008). Mesothelium contributes to vascular smooth muscle and mesenchyme during lung development. Proc. Natl. Acad. Sci. U. S. A. 105 (43), 16626–16630. 10.1073/pnas.0808649105 18922767 PMC2567908

[B34] Rehrauer H. Wu L. Blum W. Pecze L. Henzi T. Serre-Beinier V. (2018). How asbestos drives the tissue towards tumors: YAP activation, macrophage and mesothelial precursor recruitment, RNA editing, and somatic mutations. Oncogene 37 (20), 2645–2659. 10.1038/s41388-018-0153-z 29507420 PMC5955862

[B35] Ricciardi S. Cardillo G. Zirafa C. C. Carleo F. Facciolo F. Fontanini G. (2018). Surgery for malignant pleural mesothelioma: an international guidelines review. J. Thorac. Dis. 10, S285–S292. 10.21037/jtd.2017.10.16 29507797 PMC5830571

[B36] Saed G. M. Zhang W. Chegini N. Holmdahl L. Diamond M. P. (1999). Alteration of type I and III collagen expression in human peritoneal mesothelial cells in response to hypoxia and transforming growth factor-beta1. Wound Repair Regen. 7 (6), 504–510. 10.1046/j.1524-475X.1999.00504.x 10633010

[B37] Schelch K. Wagner C. Hager S. Pirker C. Siess K. Lang E. (2018). FGF2 and EGF induce epithelial-mesenchymal transition in malignant pleural mesothelioma cells via a MAPKinase/MMP1 signal. Carcinogenesis 39 (4), 534–545. 10.1093/carcin/bgy018 29635378

[B38] Shelton E. L. Galindo C. L. Williams C. H. Pfaltzgraff E. Hong C. C. Bader D. M. (2013). Autotaxin signaling governs phenotypic heterogeneity in visceral and parietal mesothelia. PLoS ONE 8 (7), e69712. 10.1371/journal.pone.0069712 23936085 PMC3723636

[B39] Shimamura Y. Tanaka J. Kakiuchi M. Sarmah H. Miura A. Hwang Y. (2022). A developmental program that regulates mammalian organ size offsets evolutionary distance. BioRxiv. 10.1101/2022.10.19.512107

[B40] Sontake V. Kasam R. K. Sinner D. Korfhagen T. R. Reddy G. B. White E. S. (2018). Wilms’ tumor 1 drives fibroproliferation and myofibroblast transformation in severe fibrotic lung disease. JCI Insight 3 (16), e121252. 10.1172/jci.insight.121252 30135315 PMC6141179

[B41] Stahl M. Tallman M. S. (2019). Acute promyelocytic leukemia (APL): remaining challenges towards a cure for all. Leukemia Lymphoma 60 (Issue 13), 3107–3115. 10.1080/10428194.2019.1613540 31842650 PMC7479633

[B42] Tan Y. Qiao Y. Chen Z. Liu J. Guo Y. Tran T. (2020). FGF2, an immunomodulatory factor in asthma and chronic obstructive pulmonary disease (COPD). Front. Cell Dev. Biol. 8, 223. 10.3389/fcell.2020.00223 32300593 PMC7142218

[B43] Travis W. D. Müller-Hermelink HK B. E. (2004). “Pathology and genetics: tumours of the lung, pleura, thymus and heart,” in WHO Classification of Tumours (International Agency for Research on Cancer). 3rd edn. Vol. 10, 1.

[B44] Weaver M. Dunn N. R. Hogan B. L. (2000). Bmp4 and Fgf10 play opposing roles during lung bud morphogenesis. Dev. Camb. Engl. 127 (12), 2695–2704. 10.1242/dev.127.12.2695 10821767

[B45] Weaver M. Yingling J. M. Dunn N. R. Bellusci S. Hogan B. L. (1999). Bmp signaling regulates proximal-distal differentiation of endoderm in mouse lung development. Dev. Camb. Engl. 126 (18), 4005–4015. 10.1242/dev.126.18.4005 10457010

[B46] Yuan T. Volckaert T. Chanda D. Thannickal V. J. De Langhe S. P. (2018). Fgf10 signaling in lung development, homeostasis, disease, and repair after injury. Front. Genet. 9, 418. 10.3389/fgene.2018.00418 30319693 PMC6167454

