# Supplementary figures and images for: FGF2 promotes the expansion of parietal mesothelial progenitor pools and inhibits BMP4-mediated smooth muscle cell differentiation
Source: Front Cell Dev Biol. 2024 Sep 23;12:1387237. doi: 10.3389/fcell.2024.1387237 (PMC11456698; doi:10.3389/fcell.2024.1387237)

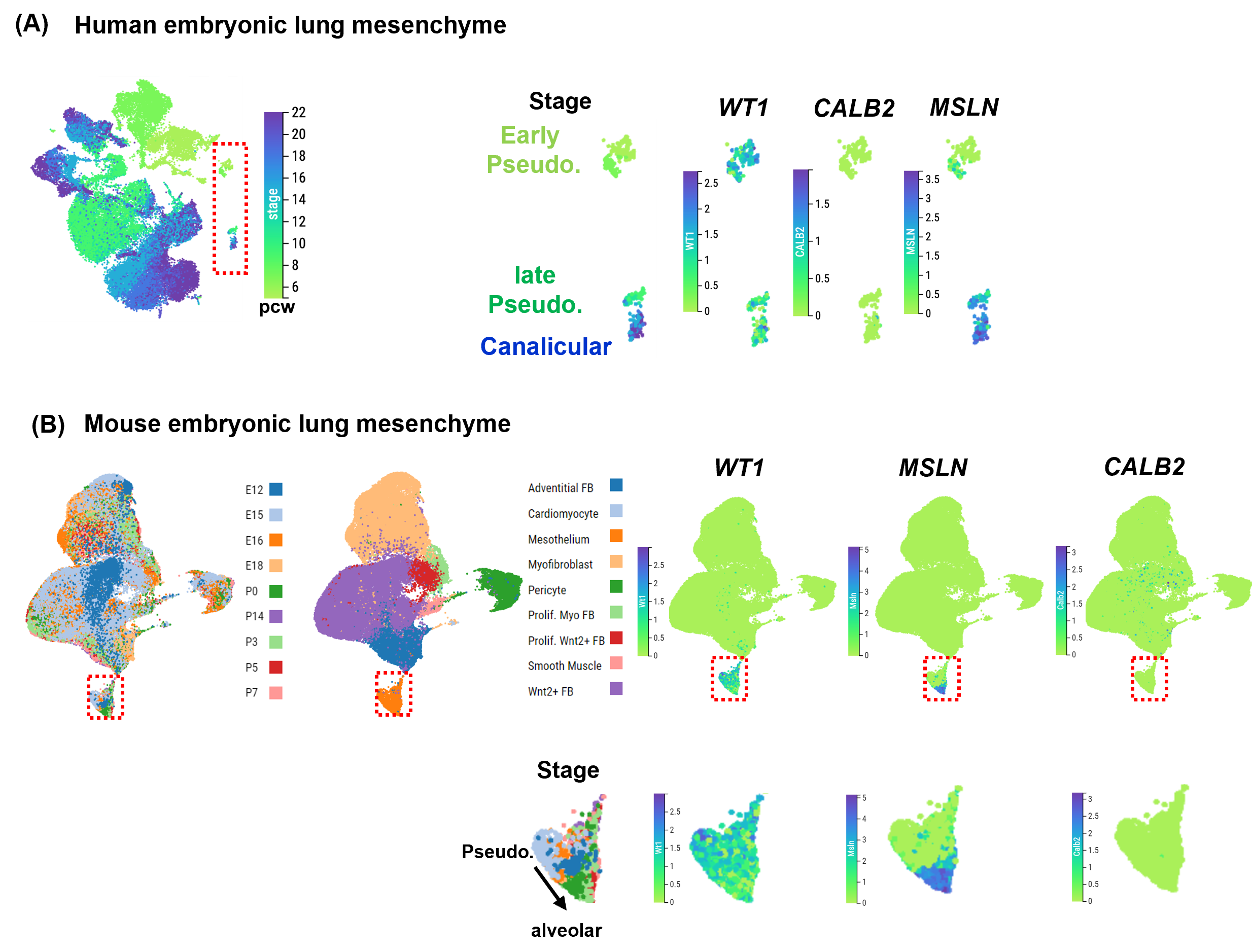

Supplement: Supplementary file 1 [file Image6.TIF]

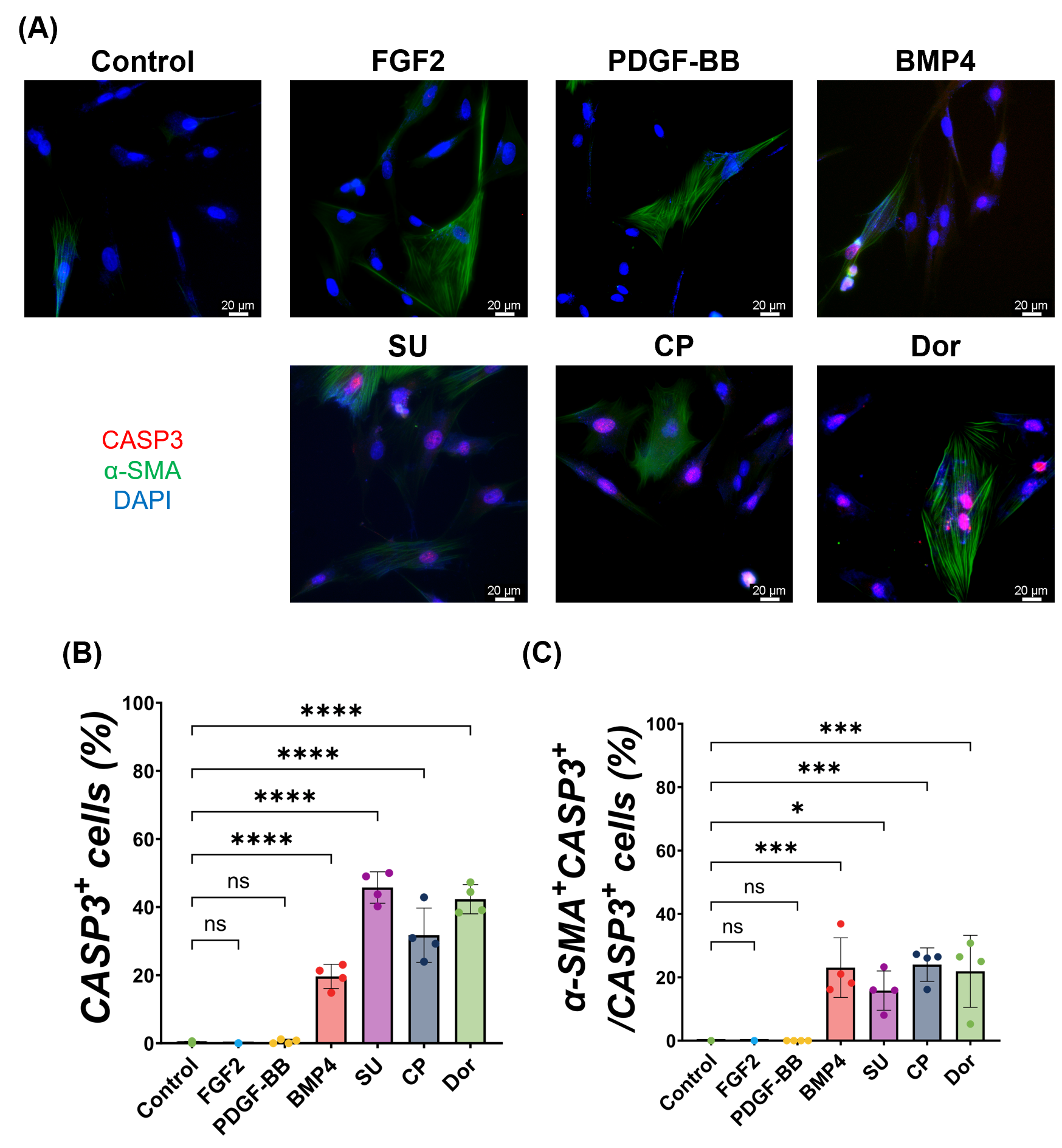

Supplement: Supplementary file 2 [file Image3.TIF]

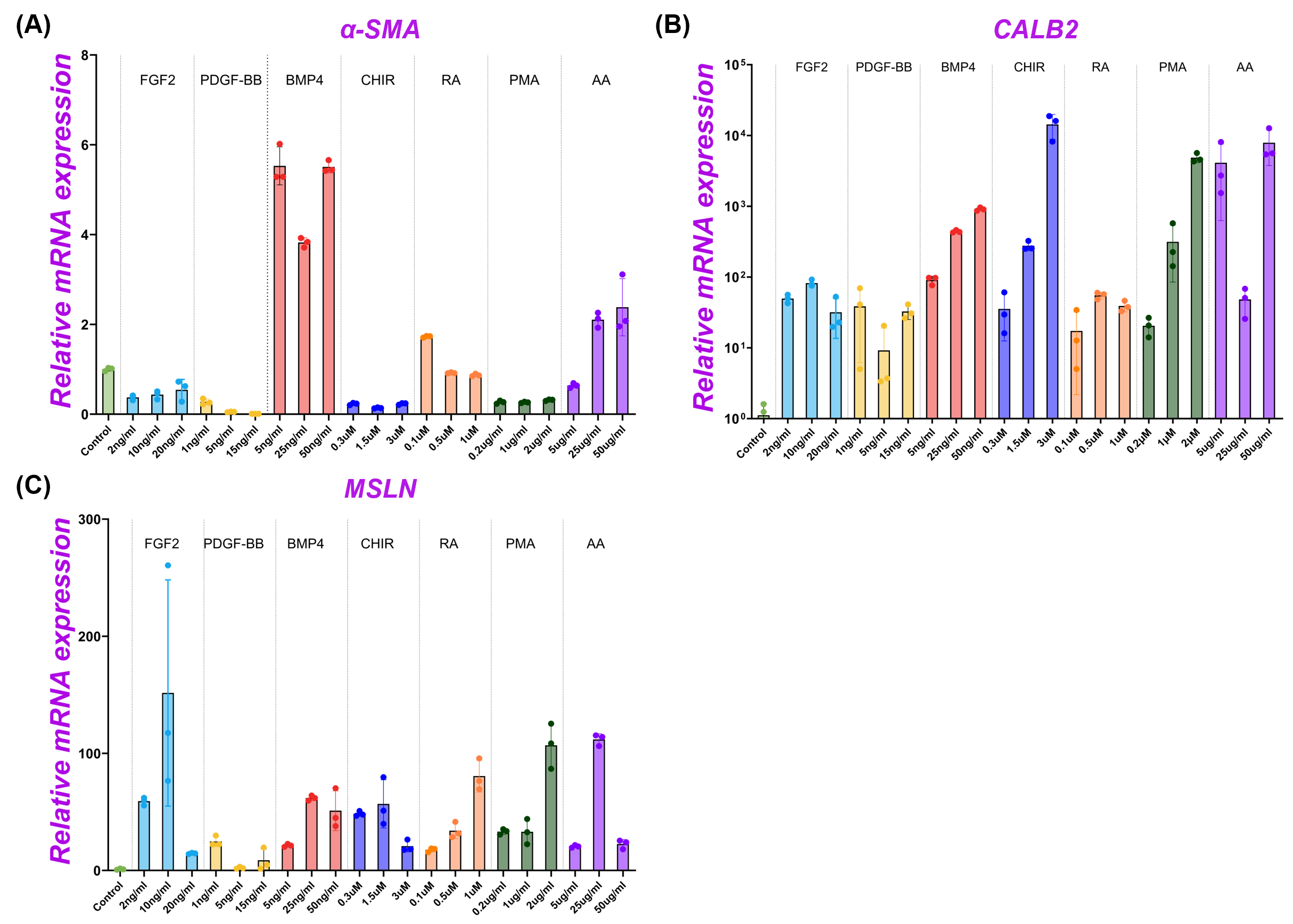

Supplement: Supplementary file 3 [file Image4.TIF]

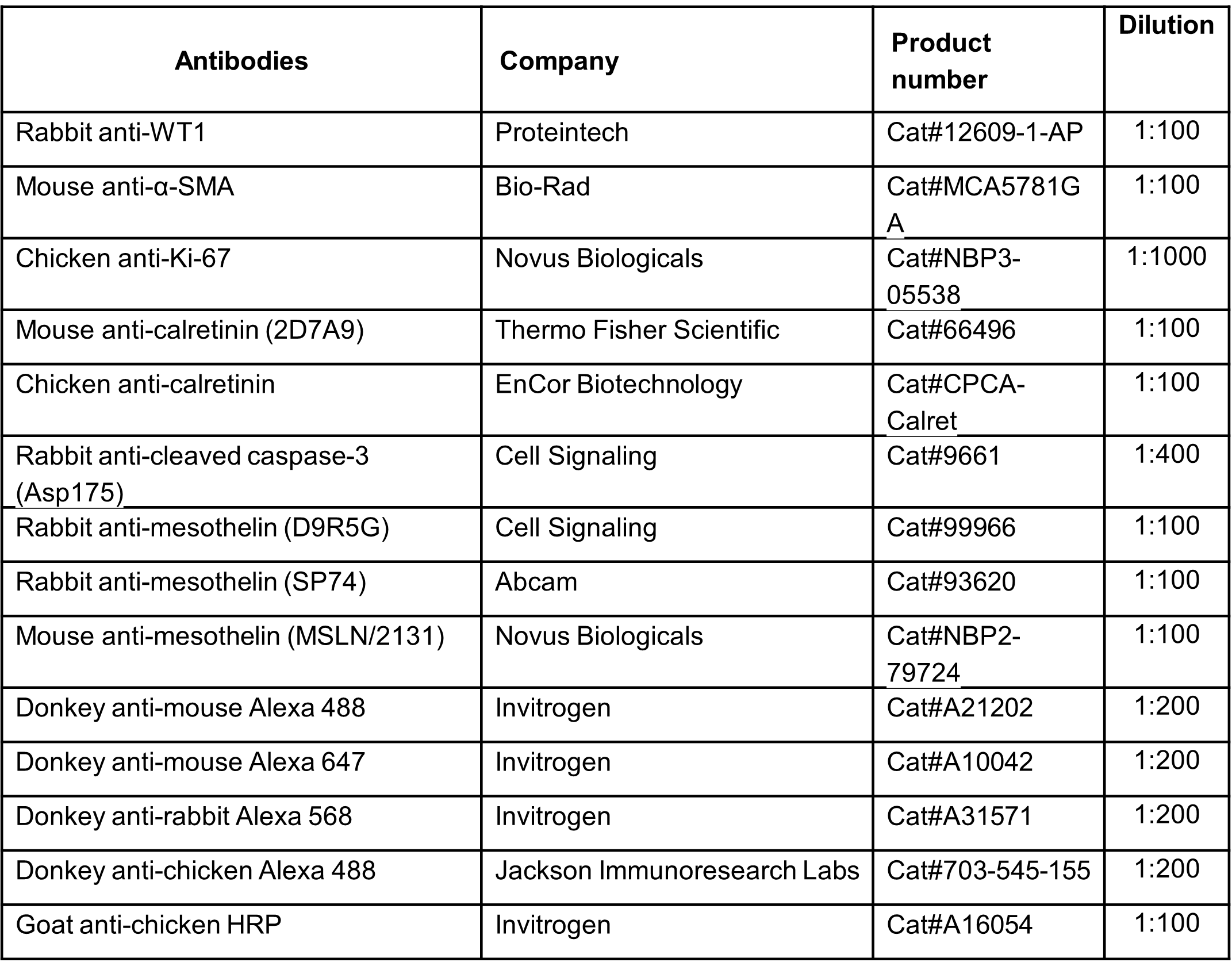

Supplement: Supplementary file 4 [file Image9.TIF]

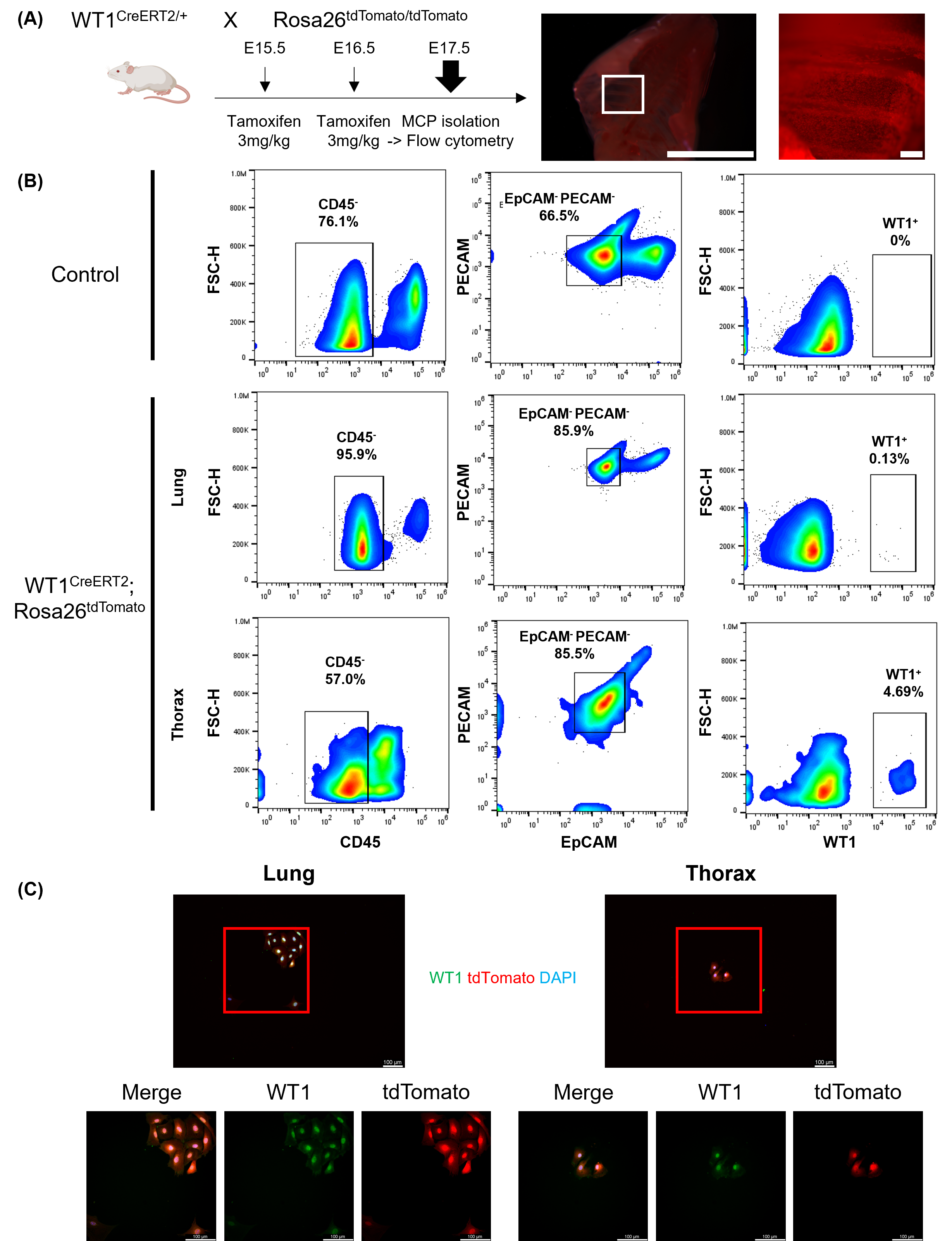

Supplement: Supplementary file 5 [file Image2.TIF]

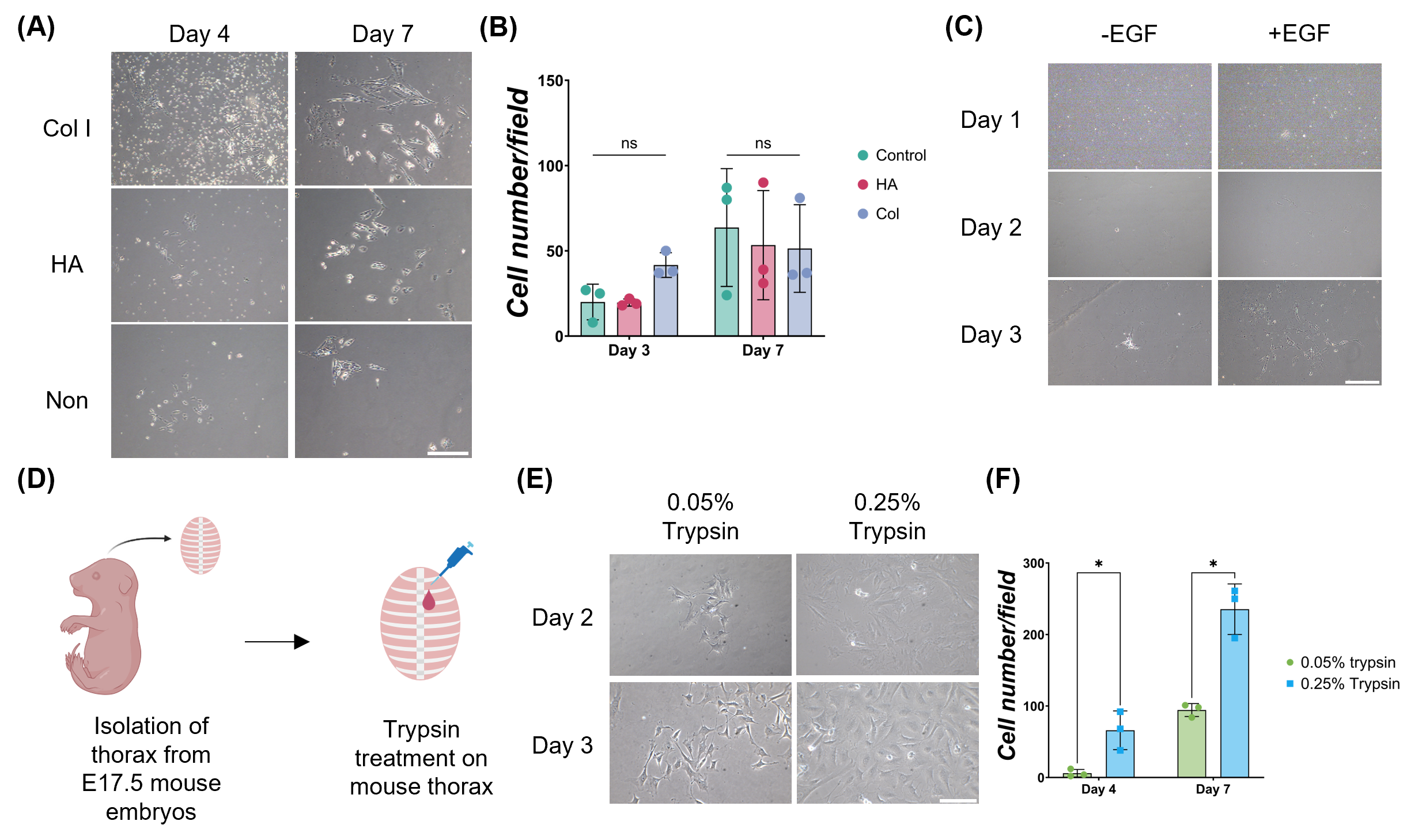

Supplement: Supplementary file 6 [file Image1.TIF]

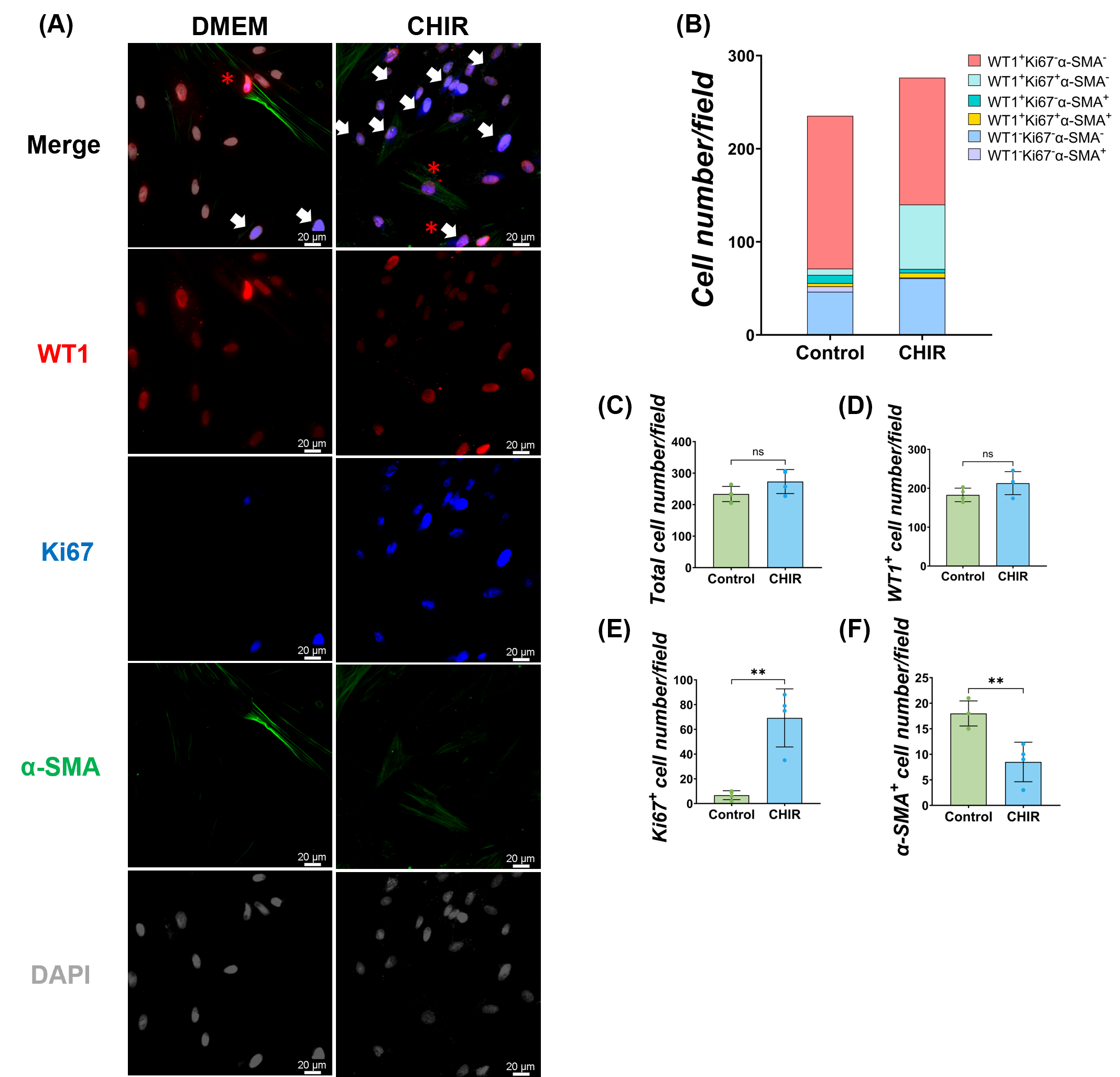

Supplement: Supplementary file 7 [file Image7.TIF]

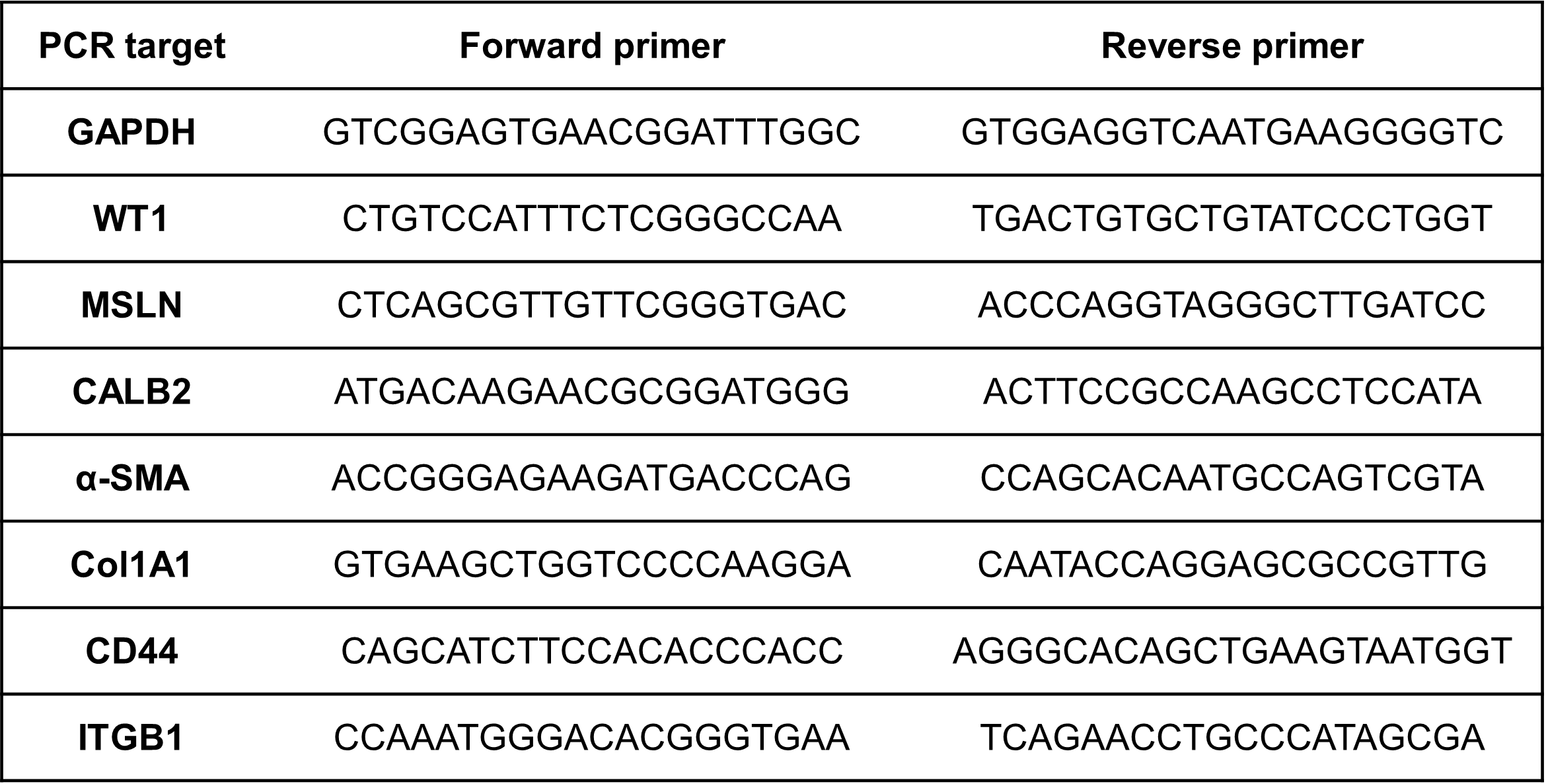

Supplement: Supplementary file 8 [file Image8.TIF]

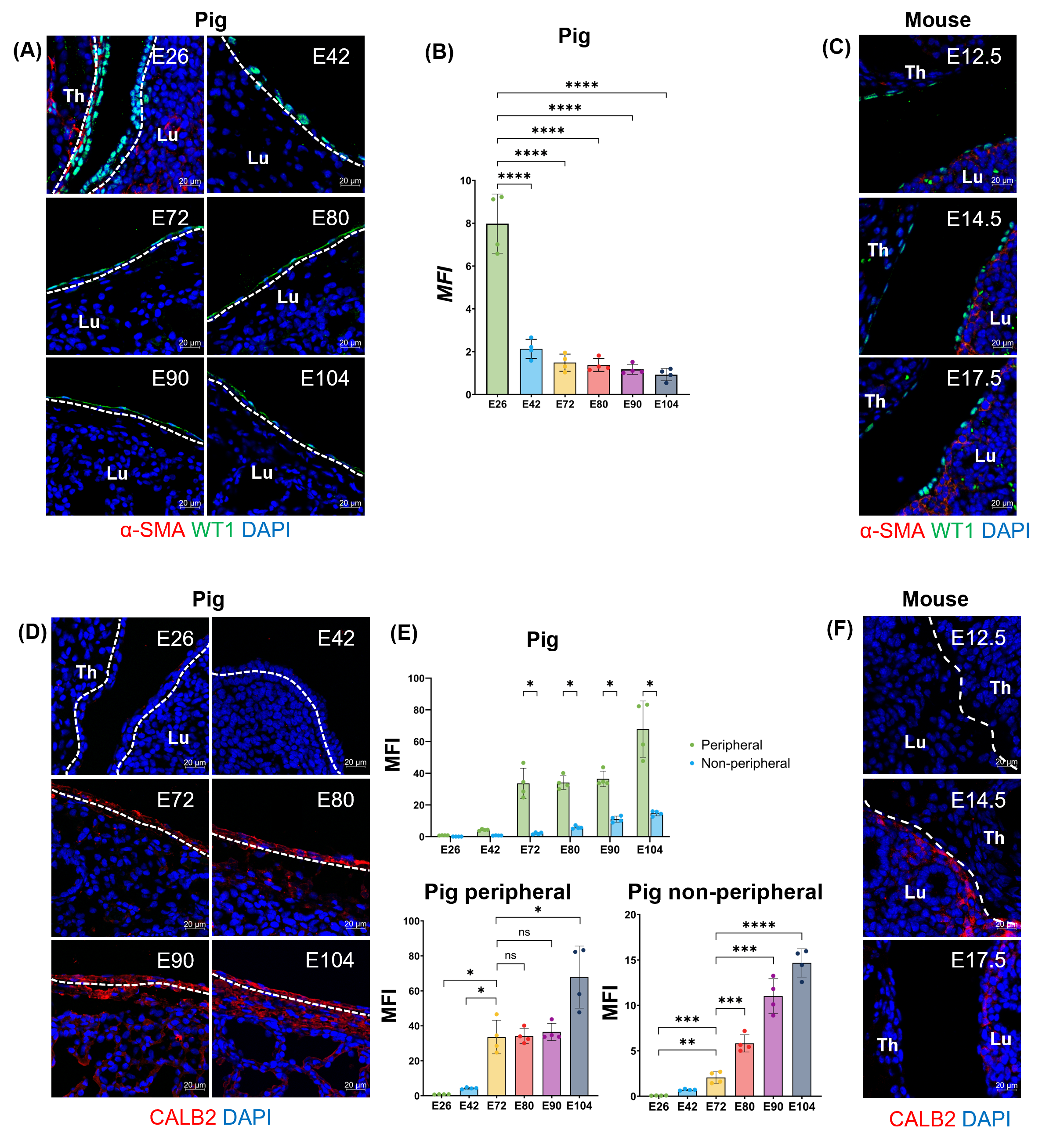

Supplement: Supplementary file 9 [file Image5.TIF]
